# Associations of spousal communication with contraceptive method use among adolescent wives and their husbands in Niger

**DOI:** 10.1371/journal.pone.0237512

**Published:** 2020-08-10

**Authors:** Sneha Challa, Holly B. Shakya, Nicole Carter, Sabrina C. Boyce, Mohamad I. Brooks, Sani Aliou, Jay G. Silverman

**Affiliations:** 1 Center on Gender Equity and Health, University of California San Diego, La Jolla, California, United States of America; 2 Pathfinder International, Boston, Massachusetts, United States of America; FHI360, UNITED STATES

## Abstract

**Objectives:**

This study aims to examine associations between spousal communication about contraception and ever use of modern contraception, overt modern contraceptive use (with husband’s knowledge), and covert modern contraceptive use (without husband’s knowledge) among adolescent wives and their husbands in Niger.

**Study design:**

Cross-sectional data, from the *Reaching Married Adolescents Study*, were collected from randomly selected adolescent wives (ages 13–19 years) and their husbands from 48 randomly selected villages in rural Niger (N = 1,020 couples). Logistic regression models assessed associations of couples’ reports of spousal communication about contraception with wives’ reports of contraception (overall, overt, and covert).

**Results:**

About one-fourth of adolescent wives and one-fifth of husbands reported spousal communication about contraception. Results showed couples’ reports of spousal communication about contraception were positively associated with ever use of modern contraception. Couples’ reports of spousal communication about contraception were negatively associated with covert modern contraceptive use compared to overt use. Wives’ reports of spousal communication were marginally associated with covert use compared to no use but husbands’ reports were not.

**Conclusion:**

Among a sample of couples in Niger, spousal communication about contraception was positively associated with modern contraceptive use (compared to no use) and negatively with covert use (compared to overt use) but wives’ and husbands’ reports showed differential associations with covert use compared to no use. Since there is little understanding of couple communication surrounding covert contraceptive use decisions, research should focus on characterizing content and context of couple communication particularly in cases of disagreement over fertility decisions.

## Introduction

In Niger, one-fourth of girls are married by age 15 years and three-fourths by age 18 years [1]. Studies have shown that child marriage can lead to negative sexual and reproductive health (SRH) outcomes for girls [2, 3]. Marriage being a central expectation of Nigerien life [4, 5], entering into a union helps both men and women fulfill their roles and thus, this practice has endured. In Hausa communities, the predominant ethnic group in Niger [6], these gender norms ascribe to women the role of bearing and raising children while men’s roles are as providers and strictly not as homemakers [5, 7, 8]. Further, social status in Hausa communities has been shown to be largely determined by gender, age, and wealth, thus placing young married girls in particularly disadvantaged positions [7]. In the context of such societal structures where inequitable gender norms prevail, male dominance over decision-making remains prevalent, preventing girls from negotiating for their SRH. Ethnographic research in the Maradi region of Niger has demonstrated the continuing hold that men have over decisions at all levels from political leadership to household assets and extending to matters of reproduction [7].

While there has been shown to be some consensus in Nigerien couples that birth spacing is important, research shows that abstinence is still seen as the most appropriate avenue and that barriers to uptake of modern contraception primarily include men’s opposition [7]. Research in sub-Saharan Africa, has demonstrated that despite these barriers, some women attempt to take control of their fertility by using contraception without their husband’s knowledge [9–12], indicating the importance of differentiating overt contraceptive users (use with husband’s knowledge) from covert contraceptive users (use without husband’s knowledge). Discreet (covert) use has been cited in Niger as well, likely due to childbearing expectations in the context of marriage [7]. Research shows that the confluence of inequitable gender norms, young age at marriage, low autonomy for young married girls, and high desired family size may all contribute to low contraceptive use [13, 14]. In Niger, this perpetuates elevated adolescent fertility rates [10] and puts married girls at uniquely high risk of adverse health outcomes.

Family planning (FP) promotion programs in sub-Saharan Africa have historically included only women, despite men often being primary decision-makers. Scholars believe that including men in these programs as FP clients, supportive partners, and potential agents of change can encourage joint decision-making around contraceptive use [15–17]. Thus, when men and women are making contraception decisions together, there may be increased utilization of reproductive health services. An example in Niger itself includes the “Schools for Husbands”, an initiative started by the United Nations Population Fund aimed at engaging men in finding solutions to SRH problems [18]. Interventions in several low- and middle- income countries have emphasized promotion of couple communication as an important modality of male engagement [17, 19, 20], arguing that support from and communication with partners will improve contraceptive use, but these studies do not focus on representative samples of married adolescent girls [21–24]. What is more, it has been shown that husbands and wives in Niger occupy entirely different spheres in society and their daily lives, potentially limiting couples’ interactions [5, 7] and comfort in communicating, particularly on sensitive topics such as contraception. Though the ‘Schools for Husbands’ in Niger have gained traction and marital relationships may have shifted as a result [18, 25], the programs are currently limited in scope and reach with little data on both husbands’ and wives’ perceptions of their relationship dynamics and the resulting dialogue regarding SRH topics. Therefore, this study aims to assess associations between adolescent wives’ and their husbands’ reports of spousal communication regarding contraception and wives’ reported actual contraceptive use in the Dosso region of Niger.

## Materials and methods

### Data source

This cross-sectional analysis utilizes baseline data from the *Reaching Married Adolescents (RMA) Study* (ClinicalTrials.gov NCT03226730), a cluster randomized control trial evaluating an FP promotion intervention among adolescent wives and their husbands in Niger. Baseline data were collected from April-June 2016 from adolescent wives ages 13–19 years and their husbands (N = 1,072) across 48 villages (12 intervention villages and 4 control villages in each of 3 districts) in the Dosso region. Villages were randomly selected based on: 1) having at least 1000 permanent inhabitants, 2) primarily Hausa or Zarma-speaking, 3) located in Dosso, Doutchi, or Loga districts (of the Dosso region), and 4) no other NGO known to be intervening specifically around FP or female empowerment with adolescent wives or their husbands. Participants were then randomly selected (using a random number generator and selecting the first 25 households) from a list of all adolescent wives provided by village chiefs. Girls were considered eligible if they were: 1) aged 13–19 years old, 2) married, 3) fluent in Hausa or Zarma, 4) residing in the village where recruitment was taking place with no plans to move away in next 18 months or plans to travel for more than 6 months during that period, and 5) not currently sterilized. More information about study design and data collection can be found in the protocol paper for this study [26].

On pre-programmed tablets, trained, gender-matched Research Assistants collected self-report data using surveys that were developed in English, translated to written French, and verbally translated to Hausa or Zarma. Surveys were conducted in a private location identified by each participant, taking approximately 45–60 minutes. Research Assistants visited selected households to confirm eligibility using a Household Recruitment Survey. If a household had no eligible participants, a replacement was randomly chosen until sample size was reached. Research Assistants introduced the study to eligible participants and, in keeping with local customs, obtained assurance from husbands (or male heads of household) for adolescent wives’ participation. Given the low literacy rate in the sample, explicit verbal consent was also obtained from each married adolescent girl. The need for parental consent was waived as this sample comprises adolescent wives who are considered emancipated due to their marital status. Encrypted and de-identified data were uploaded to a server via secure internet connection weekly. This study was approved by the Institutional Review Board of the University of California San Diego as well as the Institutional Review Board of the Nigerien Ministry of Health.

In the survey, adolescent wives were asked who had the most influence over contraceptive method use to space or delay pregnancies. Response options included husband, mother-in-law, the wife herself, husband’s brother, father-in-law, and co-wife. Over 95% reported that their husbands were their top decision-makers (N = 1,020); the remaining 52 (4.9%) were excluded from the current analyses to allow testing of hypotheses for couples who reported spousal communication, as questions related to communication about contraception for adolescent wives’ were only asked with respect to their top decision-makers.

### Measures

The exposure in this analysis was spousal communication about contraception, while the outcomes were any use of modern contraceptive methods, and the subcategories of overt modern contraceptive use and covert modern contraceptive use (all ‘ever’ due to the very low rates of contraceptive use in this sample). For all predictors and outcomes “don’t know” and decline responses were coded as missing. Only those with complete data on these measures were included. To measure spousal communication about contraception, adolescent wives were asked whether they had ever had a discussion with their top decision-makers (in this case, their husbands) about using a contraceptive method to space or delay pregnancy while husbands were asked the same question of discussions with their wives.

If wives responded “yes” to currently doing something or using any method to space or delay pregnancy or having done so in the past, they were asked what methods they had used. They were considered to have used *any* modern contraception if they reported using any of the following: IUD, injectables, implants, pills, male condoms, female condoms, emergency contraception, lactation amenorrhea method. Additionally, wives were asked if their husbands knew about this use. We defined *covert* modern contraceptive users as those who reported that their husbands were not aware that they had used contraception and *overt* modern contraceptive users as those who reported that their husbands were aware of this use.

Demographic information was collected in the Household Recruitment Survey, during which heads of household were asked about how old the wife and her husband were on their last birthdays, the wife’s age at marriage, the husband’s and wife’s educational attainment, the number of other wives married to this husband, and the wife’s number of living children. Wife’s age, age difference between husband and wife (created by subtracting wife’s age from husband’s), wife’s age at marriage, and wife’s parity were treated continuously. Education included husband’s and wife’s attendance at government school (where students learn reading/writing/math), Quranic school only (where students receive an Islamic religious education), or neither. Indicative of economic security, adolescent wife participants in the main survey were asked whether in the month prior to the interview they or any member of their family had gone without eating the whole day because there was not enough food (food insecurity). Additional demographic characteristics included male migration (whether husbands had been traveling for greater than six months) and district.

### Data analysis

All analyses were conducted using *SAS Studio*^®^ (SAS Institute Inc., 2018). Descriptive statistics were used to assess differences in demographic variables by outcome variables. For both adolescent wives’ and husbands’ reports, separate logistic regression models were used to assess associations between spousal communication about contraception and adolescent wives’ reports of any use of modern contraception (yes vs no) while associations with adolescent wives’ reports of contraceptive use with/without husbands’ knowledge were examined using multinomial regression (no use vs overt use vs covert use). First, unadjusted models were constructed with just the main exposure and outcomes of interest, producing odds ratios (ORs) and 95% confidence intervals (95% CIs). Then, adjusted models were constructed, accounting for covariates that literature has demonstrated are associated with contraceptive use as well as those found to be associated with the outcomes at p<0.05 in the first step. This set of covariates was consistent across all models.

## Results

### Characteristics of the sample

Adolescent wives were between the ages of 13–19 years with over half of the sample being 18–19 years old (n = 547, 53.6%) and almost 30% of husbands being ten or more years older. Wives’ median age at marriage was 14 and 407 (39.9%) were married between the ages of 14–15. Many adolescent wives had no formal education, with 490 (48.0%) reporting no schooling and 352 (34.5%) having attended government school. More husbands than wives had received an education: 469 (46.0%) attended government school while 308 (30.2%) received no schooling. Of the 1,020 adolescent wives in our study, 123 (12.1%) reported ever having used any modern contraception (Table 1). This comprised primarily users of pills, injectables, and implants at 6.1%, 5.0%, 1.6% of the sample respectively. Users of all other modern contraceptive methods make up less than 1% of the sample combined. Of those that had used any modern contraception, 94 (9.2% of the total sample) reported ever having used modern contraception overtly (with their husband’s knowledge) and 29 (2.8% of the total sample) reported ever having used modern contraception covertly (without their husband’s knowledge). Among adolescent wives, 26% reported ever having discussed contraception with their husbands while 20% of husbands reported these discussions. Almost 60% of couples agreed that they had never had a discussion about using any method or doing something to space or delay pregnancy but 25% of the time there were discordant reports of spousal communication about contraception with 11% of wives stating there had been no discussion and husbands stating that there had, and 14% of wives stating there had been a discussion when the husbands stated there had not.

**Table 1:** Sample demographic characteristics of adolescent wives from Dosso, Doutchi, and Loga, Niger.

	Total	Any Use of Modern Contraception		Covert or Overt Use of Modern Contraception			
	N(%)	Yes Use (n = 123) N(%)	*p-value*	No Use (n = 883) N(%)	Overt Use (n = 94) N(%)	Covert Use (n = 29) N(%)	*p-value*
**Wife's Age**							
*14 and under*	48(4.7)	2(1.6)	*<0*.*001*^a^	44(5.0)	1(1.1)	1(3.5)	<0.001^b^
*15–17*	425(41.7)	30(24.4)		390(44.2)	27(28.7)	3(10.3)	
*18 and over*	547(53.6)	91(74.0)		449(50.9)	66(70.2)	25(86.2)	
**Husband's Age**							
*15–24*	469(46.0)	39(31.7)	*<0*.*001*^a^	425(48.1)	30(31.9)	9(31.0)	<0.001^b^
*25–29*	312(30.6)	46(37.4)		258(29.2)	34(36.2)	12(41.4)	
*30 and above*	208(20.4)	35(28.5)		172(19.5)	28(29.8)	7(24.1)	
**Age Difference**							
*4 years or less*	197(19.3)	18(14.6)	*0*.*008*^a^	177(20.1)	12(12.8)	6(20.7)	0.002^b^
*5–6 years*	243(23.8)	23(18.7)		218(24.7)	19(20.2)	4(13.8)	
*7–9 years*	250(24.5)	30(24.4)		216(24.5)	23(24.5)	7(24.1)	
*10 years or more*	299(29.3)	49(39.8)		244(27.6)	38(40.4)	11(37.93)	
**Wife's Age at Marriage**							
*13 and under*	373(36.6)	60(48.8)	*<0*.*001*^a^	307(34.8)	50(53.2)	10(34.5)	<0.001^b^
*14–15*	407(39.9)	42(34.2)		360(40.8)	33(35.1)	9(31.0)	
*16–17*	203(19.9)	20(16.3)		180(20.4)	11(11.7)	9(31.0)	
*18–19*	34(3.3)	0(0.0)		34(3.9)	0(0.0)	0(0.0)	
**Parity**							
*No Children*	402(39.4)	5(4.1)	*<0*.*001*^a^	390(44.2)	1(1.1)	4(13.8)	*<0*.*001*^b^
*1 Child*	340(33.3)	46(37.4)		290(32.8)	33(35.1)	13(44.8)	
*2 Children or More*	278(27.3)	70(58.5)		203(23.0)	60(63.8)	12(41.4)	
**Wife's Education**							
*Government School*	352(34.5)	41(33.3)	*0*.*15*^c^	303(34.3)	35(37.2)	6(20.7)	*0*.*083*^c^
*Quranic School*	169(16.6)	27(2.0)		138(15.6)	22(23.4)	5(17.2)	
*No School*	490(48.0)	52(42.3)		436(49.4)	35(37.2)	17(58.6)	
**Husband's Education**							
*Government School*	469(46.0)	56(45.5)	*0*.*004*^c^	404(47.5)	42(44.7)	14(48.3)	*0*.*017*^c^
*Quranic School*	207(20.3)	38(30.9)		168(19.0)	31(33.0)	7(24.1)	
*No School*	308(30.2)	26(21.1)		278(31.5)	19(20.2)	7(24.1)	
**Number of Wives**							
*Monogamous*	858(84.1)	105(85.4)	*0*.*80*^c^	741(83.9)	81(86.2)	24(82.8)	*0*.*92*^c^
*Polygamous*	131(12.8)	15(12.2)		114(12.9)	11(11.7)	4(13.8)	
**Food Insecurity**							
*No*	789(77.4)	89(72.4)	*0*.*16*^c^	687(78.8)	68(72.3)	21(72.4)	*0*.*37*^c^
*Yes*	228(22.4)	34(27.64		193(21.9)	26(27.7)	8(27.6)	
**Has husband spend >3 months away**							
*No*	300(29.4)	35(28.5)	*0*.*79*^c^	258(29.2)	27(28.7)	8(27.6)	*0*.*96*^c^
*Yes*	684(67.1)	85(69.1)		592(67.0)	65(69.2)	20(69.0)	
**Predictor**							
**Adolescent Wives’ Reports of Communication**							
*No*	750(73.5)	34(27.6)	*<0*.*001*^c^	711(80.5)	15(16.0)	19(65.5)	*<0*.*001*^c^
*Yes*	262(25.7)	89(72.4)		166(18.8)	79(84.0)	10(34.5)	
**Husbands’ Reports of Communication**							
*No*	740(72.6)	66(53.7)	*<0*.*001*^c^	665(75.3)	44(46.8)	22(75.9)	*<0*.*001*^c^
*Yes*	207(20.3)	46(37.4)		157(17.8)	42(44.7)	4(13.8)	

^a^p-value for one-sample t-tests comparing 2-category outcome (any use/no use) across demographic characteristics (continuous).

^b^p-value for ANOVAs comparing 3-category outcome (cover use/overt use/no use) across demographic characteristics (continuous).

^c^p-values for chi-square tests for outcomes across demographic characteristics (categorical).

### Spousal communication and contraceptive use

Among the 123 (12.1% of total) that had used any modern contraception 89 wives (72.4%) reported spousal communication about contraception while 46 husbands (37.4%) did so. For wives, within the categories of overt and covert modern contraceptive use, 79 (84.0%) and 10 (34.5%) respectively, reported spousal communication about contraception while 166 (18.8%) of never users reported spousal communication about contraception. Of the husbands, 44.7% were married to wives who had used contraception overtly while 13.8% were married to wives that had used covertly. A similar proportion of husbands of never users (17.8%) reported such communication. Results examining associations between demographic characteristics and spousal communication about contraception in a multivariable model (S1 Table) showed that adolescent wives had lower odds of spousal communication about contraception as age of marriage increased (AOR: 0.89, 95% CI: 0.80, 0.98, p = 0.02) but higher odds of communication about contraception as age increased (AOR: 1.23, 95% CI: 1.07, 1.41, p = 0.004), as number of children increased (AOR: 1.43, 95% CI: 1.16, 1.77, p<0.001), and if their husbands attended government school compared to no school (AOR: 1.77, 95% CI: 1.18, 2.63, p = 0.005). Similarly, husbands’ reports of communication (S2 Table) were also associated negatively with their wives’ age at marriage (AOR: 0.88, 95% CI: 0.78, 0.98, p = 0.02) but positively associated with parity (AOR: 1.36, 95% CI: 1.09, 1.69, p = 0.007). Husbands’ attendance at Quranic school was associated with their own reports of spousal communication about contraception (AOR: 1.70, 95% CI: 1.04, 2.76, p = 0.05), while their attendance at government school was marginally associated (AOR: 1.41, 95% CI: 0.93, 2.14, p = 0.10).

In unadjusted models (Table 2), wives’ reported spousal communication about contraceptive use was positively associated with any use of modern contraception (OR: 11.21; 95% CI: 7.30, 17.23, p<0.001) as were husbands’ reports (AOR: 2.92, 95% CI: 1.95, 4.47, p<0.001). This relationship remained after adjusting for relevant covariates for wives (OR: 8.85, 95% CI: 5.48, 14.27, p<0.001) and for husbands (AOR: 2.14, 95% CI: 1.36, 3.38, p = 0.001). Further, in unadjusted models, wives’ reported spousal communication about contraception was positively associated with overt use compared to no use (OR: 22.55, 95% CI: 12.66, 40.17, p<0.001) and covert using compared to no use, (OR: 2.25, 95% CI: 1.03, 4.94, p = 0.04) but negatively associated with covert use compared to overt use (OR: 0.10, 95% CI: 0.04, 0.26, p<0.001). After adjusting for relevant covariates, wives’ reported spousal communication about contraception was positively associated with overt use compared to no use (AOR: 16.83, 95% CI: 9.06, 31.24, p<0.001) and marginally positively associated with covert use compared to no use (AOR: 2.07, 95% CI: 0.87, 4.891, p = 0.10). Again, wives’ reported spousal communication about contraception was negatively associated with covert use compared to overt use (AOR: 0.12, 95% CI: 0.04, 0.34, p<0.001). A similar pattern was seen for husbands’ reports of communication whereby they were positively associated with overt use vs no use in both unadjusted (AOR: 4.04, 95% CI: 2.56, 6.39, p<0.001) and adjusted models (AOR: 2.74, 95% CI: 1.66, 4.53, p<0.001). Husbands’ reports of spousal communication about contraception were also negatively associated with covert use vs overt use in unadjusted (AOR: 0.19, 95% CI: 0.06, 0.60, p = 0.005), and adjusted models (AOR: 0.26, 95% CI: 0.08, 0.84, p = 0.03). However, husband’s reports were not associated with covert use vs no use.

**Table 2:** Unadjusted and adjusted associations of communication with use of modern contraception (Any, Covert, Overt).

	Any Use vs No Use		Overt Use vs No Use		Covert Use vs No Use		Covert use vs Overt Use	
	Crude	Adjusted^a^	Crude	Adjusted^a^	Crude	Adjusted^a^	Crude	Adjusted^a^
	OR	AOR	AOR	AOR	AOR	AOR	AOR	AOR
	(95% CI)	(95% CI)	(95% CI)	(95% CI)	(95% CI)	(95% CI)	(95% CI)	(95% CI)
	p-value	p-value	p-value	p-value	p-value	p-value	p-value	p-value
**Adolescent Wives’ Reported Spousal Communication**								
*No Communication*	ref	ref	ref	ref	ref	ref	ref	ref
*Yes Communication*	11.21	8.85	22.55	16.83	2.25	2.07	0.10	0.12
	(7.30, 17.23)	(5.48, 14.27)	(12.66, 40.17)	(9.06, 31.24)	(1.03, 4.94)	(0.87, 4.91)	(0.04, 0.26)	(0.04, 0.34)
	*<0*.*001*	*<0*.*001*	*<0*.*001*	<0.001	*0*.*04*	*0*.*10*	*<0*.*001*	*<0*.*001*
**Husbands’ Reported Spousal Communication**								
*No Communication*	ref	ref	ref	ref	ref	ref	ref	ref
*Yes Communication*	2.92	2.14	4.04	2.74	0.77	0.71	0.19	0.26
	(1.95, 4.47)	(1.36, 3.38)	(2.56, 6.39)	(1.66, 4.53)	(0.26, 2.27)	(0.23, 2.16)	(0.06, 0.60)	(0.08, 0.84)
	<0.001	*0*.*001*	*<0*.*001*	*<0*.*001*	*0*.*64*	*0*.*54*	*0*.*005*	*0*.*03*

^a^Covariates: wife’s age, husband-wife age difference, wife’s age at marriage, parity, wife’s education, husband’s education, district.

## Discussion

The aim of this analysis was to examine associations of spousal communication about contraception with ever having used modern contraception (any modern method use, overt modern method use, covert modern method use). In our sample, of the small proportion that reported ever having used any modern contraception to space or delay pregnancy, a majority had done so overtly (with their husband’s knowledge) as compared to covertly (without husband’s knowledge). In this case, contraceptive use was slightly below prevalence seen in the West Africa region between 2010–2015 [27]. Specifically, overall use is very low among the youngest and nulliparous, increasing with both age and parity. This pattern is in keeping with local cultural practices whereby it is expected that, regardless of age, young married women prove their fertility before contraceptive use is seen as acceptable, even by health providers [28]. What is more, we see that more women who have husbands who migrate for extended periods of time report use of modern contraception. As economic insecurity continues to threaten the livelihoods of rural Nigeriens, more are forced to leave their natal homes in search of work [29]. As a result, women in these households may be more likely to desire delaying, spacing, or limiting childbirth using modern contraceptive methods.

An important finding is that only about one-fourth of adolescent wives in our sample reported ever having discussed contraception with their husbands and one-fifth of husbands reported these discussions, a prevalence lower than found in relevant recent studies in sub-Saharan Africa [22, 30–32]. While research has shown that Nigerien women have in recent times exercised some amount of agency in FP [7], significant and persistent gender inequality [33] may bolster traditional gender roles, playing a large part in determining engagement in communication behaviors. These power dynamics, in part, may explain why a slightly smaller proportion of husband reported having discussions with their wives about contraception than adolescent wives reporting discussions with their husbands. In 14% of couples in our sample, wives stated there had been discussions about contraception but husbands did not. There is ethnographic evidence in Niger that as wives gain access to knowledge about family planning and contraceptive methods themselves, men begin to feel their status in the household is threatened [7]. Under these circumstances, it is possible that husbands state their opinions or intentions regarding FP unequivocally such that he does not consider it a discussion and simply an exertion of his rights but she perceives it as a discussion in which her husband has stated his disapproval.

In this sample, wife’s age, parity, and husband’s attendance at government school were positively associated with ever having had a discussion about contraception while age at marriage was negatively associated. While increased age, and number of children are often linked to contraceptive use and related behaviors [34], offering women increased social standing, husband’s educational attainment may foster attitudes more supportive of contraception or gender equity allowing their wives to feel more able to participate in such discussions. It is possible however, that in a culture where early marriage and childbearing are normative, when girls are married at older ages, they face even more pressure to prove their fertility, precluding couples from engaging in communication about contraception when girls are married later. It is also possible that because these marriages have been shorter in duration, wives do not yet have the freedom to drive these discussions—this lack of agency has been shown to be particularly true in the early years of marriage [7]. Husbands’ demographics followed a similar pattern of association with their own reports of communication about contraception, except that their wives’ ages were not associated and attendance at Quranic school was positively associated. There is work that supports the latter finding, demonstrating that there is belief that Islam and teachings at Quranic school support birth spacing [7, 35].

Results showed that for both wives and husbands, spousal communication about contraception was positively associated with ever having used any modern contraception. Findings also showed that wives’ reports of spousal communication about contraception were marginally significantly associated with higher odds of covert contraceptive use compared to non-use but that this was not true for husbands’ reports of communication. Due to the small number of covert users, it is difficult to draw strong conclusions about this. However, descriptively, a smaller proportion of husbands reported communication when their wives reported covert use compared to the proportion of wives that reported both communication and covert use. It is possible that this covert use is occurring in couples in which husbands expressed their disapproval of contraception which wives viewed as communication but husbands did not, viewing it instead as a statement of their opinions or beliefs. Additionally, spousal communication about contraception reported by both wives and husbands was associated with lower odds of covert use compared to overt use. Perhaps for a married adolescent girl to have decided to use a contraceptive method covertly, both husband and wife saw their interaction as communication, but this communication stemmed from or resulted in disagreement regarding contraception. Conversely, a married adolescent girl’s open or overt use of contraception may have been due to communication where her husband was supportive or where both husband and wife agreed on contraceptive use. Further research is needed to understand adolescent wives’ decisions to use contraception covertly and the relationship dynamics that may contribute to these decisions. Relationship conflict particularly, may play a role in determining how couple communication affects contraceptive use decisions, and merits further exploration, particularly because research has shown that girls married early are at higher risk of adverse relationship experiences such as intimate partner violence [36].

The current findings regarding spousal communication about contraception among couples in which the wife is an adolescent, a population particularly vulnerable to early childbirth and maternal mortality, contribute to the growing literature on couple communication and reproductive health in sub-Saharan Africa and other low and middle-income country contexts. While work has demonstrated the link between couple communication about SRH topics and actual contraceptive use in low resource settings [22–24, 37, 38], there is limited focus on adolescent wives in rural West Africa. With the increasing popularity of male engagement interventions currently in the SRH field and the emphasis on early marriage prevention, our findings provide additional information regarding the relationship dynamics of married girls and their husbands. As couple-based and male engagement programs such as UNFPA’s ‘Schools for Husbands’ take root and expand across Niger and the region, we hope that our work broadens the knowledge base, supporting not only continued need for engaging couples in open communication but also offering insight into how to encourage couple communication to effectively promote women-led or joint decision-making, ultimately boosting women’s health.

The strengths of our study lie in its use of dyadic data which allow for unique perspectives on relationships from both members of the couple. Additionally, inclusion of data from adolescent wives in Niger is critical as they are an underserved and at-risk population about whom little is known. Limitations include the cross-sectional nature of these data (due to which we cannot ascertain the temporality of variables studied nor can we establish causality), and our reliance on self-report measures of sensitive information which produce risks of social desirability bias. Additionally, because Niger is a Francophone country and Hausa and Zarma are the local languages (both oral not written languages) it is possible that the questions may have changed meaning in the two-step translation process (English to French to Hausa/Zarma). Eligibility criteria included adolescent wives being between ages 13–19 years, with recruitment taking place by random selection from a list of all adolescent wives in each village but our sample was concentrated towards the high end of this age range. Anecdotal evidence from in-country partners suggested that for many of the communities involved in our study, this was the first time an FP promotion program had come to them seeking to include adolescents. Due to the belief that contraception is not for younger women, village chiefs who provided lists of adolescent wives for recruitment may have included mostly older adolescents (Personal Communication, Dr. Sani Aliou, June 9, 2020). This is supported by qualitative research that indicates a lack of support for contraceptive use before fertility has been proven or before fertility goals have been achieved [7, 39]. Future research should pay particular attention to the social norms that preclude the youngest girls from engaging in FP programs and contraceptive use and more broadly in community life. While our findings are important for knowledge of couple communication in this context, we only measured presence or absence of communication regarding contraception—but not the contexts, motivations for, or responses to this communication. In the future, significant efforts should be made to understand the reasons for and results of couple communication, particularly the relationship context that leads to decisions to use contraception covertly and the content of communication in the presence of disagreement within married couples.

## Conclusion

In our study, wives’ and husbands’ reports of spousal communication about contraceptive use were positively associated with ever having used a modern contraceptive method to space or delay pregnancy but negatively associated with covert use compared to overt use among a sample of married girls and their husbands in three districts of the Dosso region of Niger. Future research on couple communication should focus on characterizing the content and context of these communications and the resulting contraceptive use decisions, particularly with respect to covert use. Findings from this work are critical to informing efforts to engage men partnered with adolescent girls regarding fertility and reproductive health decisions.

## Supporting information

S1 Table Multivariable model of wives reports of spousal communication about contraception with all covariates. (DOCX)

S2 Table Multivariable model of husbands’ reports of spousal communication about contraception with all covariates. (DOCX)

S1 File Female survey. (DOCX)

S2 File Male survey. (DOCX)

S3 File Household recruitment survey. (DOCX)

## References

[1] United Nations Children’s Fund (US). State of the World’s Chidlren. New York, NY: UNICEF, 2016.

[2] Santhya KG. Early marriage and sexual and reproductive health vulnerabilities of young women: a synthesis of recent evidence from developing countries. Current opinion in obstetrics & gynecology. 2011;23(5):334–9. Epub 2011/08/13. 10.1097/GCO.0b013e32834a93d2 . 21836504

[3] Delprato M, Akyeampong K. The Effect of Early Marriage Timing on Women's and Children's Health in Sub-Saharan Africa and Southwest Asia. Annals of global health. 2017;83(3–4):557–67. Epub 2017/12/10. 10.1016/j.aogh.2017.10.005 . 29221529

[4] Saunders M. Marriage and divorce in a Hausa town (Mirria, Niger Republic). Bloomington, UN: Indiana University; 1978.

[5] Masquelier A. The scorpion’s sting: youth, marriage and the struggle for social maturity in Niger. Journal of the Royal Anthropological Institute. 2005;11(1). 10.1111/j.1467-9655.2005.00226.x.

[6] Central Intelligence Agency. Niger Washington, D.C.: United States Central Intelligence Agency; [cited 2020 June 9]. https://www.cia.gov/library/publications/the-world-factbook/geos/ng.html.

[7] Perlman D, Chaibou S. Women’s empowerment and resilience in Niger: An ethnographic study. Washington, D.C: Mitchell Group, 2018.

[8] Perlman D, Adamu F, Wodon Q. Understanding and ending child marriage: Insights from Hausa communities. Washington, D.C: The World Bank, 2017.

[9] Balogun O, Adeniran A, Fawole A, Adesina K, Aboyeji A, Adeniran P. Effect of Male Partner's Support on Spousal Modern Contraception in a Low Resource Setting. Ethiopian journal of health sciences. 2016;26(5):439–48. Epub 2017/04/28. 10.4314/ejhs.v26i5.5 . 28446849 PMC5389058

[10] Baiden F, Mensah GP, Akoto NO, Delvaux T, Appiah PC. Covert contraceptive use among women attending a reproductive health clinic in a municipality in Ghana. BMC women's health. 2016;16:31 Epub 2016/06/09. 10.1186/s12905-016-0310-x . 27266263 PMC4893877

[11] Gasca NC, Becker S. USING COUPLES' DISCORDANT REPORTS TO ESTIMATE FEMALE COVERT USE OF MODERN CONTRACEPTION IN SUB-SAHARAN AFRICA. Journal of biosocial science. 2018;50(3):326–46. Epub 2017/07/20. 10.1017/S0021932017000256 . 28720152

[12] Heck CJ, Grilo SA, Song X, Lutalo T, Nakyanjo N, Santelli JS. "It is my business": A Mixed-Methods Analysis of Covert Contraceptive Use among Women in Rakai, Uganda. Contraception. 2018 Epub 2018/03/08. 10.1016/j.contraception.2018.02.017 . 29514043 PMC6041694

[13] Sedgh G, Ashford LS, Hussain R. Unmet need for contraception in developing countries: examining women’s reasons for not using a method. New York, NY: Guttmacher Institute, 2016.

[14] Adams MK, Salazar E, Lundgren R. Tell them you are planning for the future: gender norms and family planning among adolescents in northern Uganda. International journal of gynaecology and obstetrics: the official organ of the International Federation of Gynaecology and Obstetrics. 2013;123 Suppl 1:e7–10. Epub 2013/09/03. 10.1016/j.ijgo.2013.07.004 . 23992625

[15] Increasing contraceptive use in Niger final report. San Francisco, CA; Seattle, WA: Camber Collective, 2015.

[16] Adamou B IB, Agala CBO, Mejia C. Male engagement in family planning: Gaps in monitoring and evaluation. Chapel Hill, NC: Measure Evaluation, 2017. 10.12688/gatesopenres.12927.1 PMC6556759 31259315

[17] Raj A, Ghule M, Ritter J, Battala M, Gajanan V, Nair S, et al Cluster Randomized Controlled Trial Evaluation of a Gender Equity and Family Planning Intervention for Married Men and Couples in Rural India. PloS one. 2016;11(5):e0153190 Epub 2016/05/12. 10.1371/journal.pone.0153190 . 27167981 PMC4864357

[18] United Nations Population Fund (US). ‘Schools for husbands’ encourages Nigerien men to improve the health of their families New York, NY: UNFPA; 2011 [cited 2020 April 20]. https://www.unfpa.org/news/‘school-husbands’-encourages-nigerien-men-improve-health-their-families.

[19] Tilahun T, Coene G, Temmerman M, Degomme O. Couple based family planning education: changes in male involvement and contraceptive use among married couples in Jimma Zone, Ethiopia. BMC public health. 2015;15:682 Epub 2015/07/22. 10.1186/s12889-015-2057-y . 26194476 PMC4509724

[20] Hartmann M, Gilles K, Shattuck D, Kerner B, Guest G. Changes in couples' communication as a result of a male-involvement family planning intervention. Journal of health communication. 2012;17(7):802–19. Epub 2012/05/02. 10.1080/10810730.2011.650825 . 22545820

[21] Uddin J, Hossin MZ, Pulok MH. Couple's concordance and discordance in household decision-making and married women's use of modern contraceptives in Bangladesh. BMC women's health. 2017;17(1):107 Epub 2017/11/11. 10.1186/s12905-017-0462-3 . 29121901 PMC5680601

[22] Prata N, Bell S, Fraser A, Carvalho A, Neves I, Nieto-Andrade B. Partner Support for Family Planning and Modern Contraceptive Use in Luanda, Angola. African journal of reproductive health. 2017;21(2):35–48. Epub 2018/04/07. 10.29063/ajrh2017/v21i2.5 . 29624938

[23] Irani L, Speizer IS, Fotso JC. Relationship characteristics and contraceptive use among couples in urban kenya. International perspectives on sexual and reproductive health. 2014;40(1):11–20. Epub 2014/04/16. 10.1363/4001114 . 24733057 PMC4317354

[24] Mostafa Kamal SM. Childbearing and the use of contraceptive methods among married adolescents in Bangladesh. The European journal of contraception & reproductive health care: the official journal of the European Society of Contraception. 2012;17(2):144–54. Epub 2012/01/17. 10.3109/13625187.2011.646014 . 22242676

[25] United Nations Population Fund (US). Schools for husbands gaining ground in rural Niger New York, NY: UNFPA; 2014 [cited 2020 April 20]. https://www.unfpa.org/news/schools-husbands-gaining-ground-rural-niger.

[26] Challa S, DeLong SM, Carter N, Johns N, Shakya H, Boyce SC, et al Protocol for cluster randomized evaluation of reaching married adolescents—a gender-synchronized intervention to increase modern contraceptive use among married adolescent girls and young women and their husbands in Niger. Reproductive health. 2019;16(1):180 Epub 2019/12/20. 10.1186/s12978-019-0841-3 . 31852538 PMC6921454

[27] Tsui AO, Brown W, Li Q. Contraceptive Practice in Sub-Saharan Africa. Popul Dev Rev. 2017;43(Suppl 1):166–91. Epub 2017/10/31. 10.1111/padr.12051 . 29081552 PMC5658050

[28] Brooks MI, Johns NE, Quinn AK, Boyce SC, Fatouma IA, Oumarou AO, et al Can community health workers increase modern contraceptive use among young married women? A cross-sectional study in rural Niger. Reproductive health. 2019;16(1):38 Epub 2019/03/27. 10.1186/s12978-019-0701-1 . 30909942 PMC6434879

[29] Gilliard P. L’extrême pauvreté au Niger: mendier ou mourir? Paris, FR: La Maison d’Edition Karthala; 2005.

[30] Dona A, Abera M, Alemu T, Hawaria D. Timely initiation of postpartum contraceptive utilization and associated factors among women of child bearing age in Aroressa District, Southern Ethiopia: a community based cross-sectional study. BMC public health. 2018;18(1):1100 Epub 2018/09/08. 10.1186/s12889-018-5981-9 . 30189842 PMC6127901

[31] Tilahun T, Coene G, Temmerman M, Degomme O. Spousal discordance on fertility preference and its effect on contraceptive practice among married couples in Jimma zone, Ethiopia. Reproductive health. 2014;11:27 Epub 2014/04/09. 10.1186/1742-4755-11-27 . 24708827 PMC3983854

[32] Wuni C, Turpin CA, Dassah ET. Determinants of contraceptive use and future contraceptive intentions of women attending child welfare clinics in urban Ghana. BMC public health. 2017;18(1):79 Epub 2017/08/03. 10.1186/s12889-017-4641-9 . 28764670 PMC5539629

[33] United Nations Development Programme (US). Niger Human Development Indicators New York, NY: UNDP; 2018 [cited 2020 April 20]. http://hdr.undp.org/en/countries/profiles/NER.

[34] Yaya S, Uthman OA, Ekholuenetale M, Bishwajit G. Women empowerment as an enabling factor of contraceptive use in sub-Saharan Africa: a multilevel analysis of cross-sectional surveys of 32 countries. Reproductive health. 2018;15(1):214 Epub 2018/12/24. 10.1186/s12978-018-0658-5 . 30572927 PMC6302468

[35] Spindler E, Perlman D, Chaibou S, Silverman J, Carter N, Boyce S, et al. Child marriage, fertility and family planning in Niger: Results from a study inspired by the International Men and Gender Equality Survey (IMAGES). Washington, D.C.: 2019.

[36] Kidman R. Child marriage and intimate partner violence: a comparative study of 34 countries. International journal of epidemiology. 2017;46(2):662–75. Epub 2016/10/14. 10.1093/ije/dyw225 . 27733435

[37] Najafi-Sharjabad F, Rahman HA, Hanafiah M, Syed Yahya SZ. Spousal communication on family planning and perceived social support for contraceptive practices in a sample of Malaysian women. Iranian journal of nursing and midwifery research. 2014;19(7 Suppl 1):S19–27. Epub 2014/02/01. . 25949248 PMC4402996

[38] Link CF. Spousal communication and contraceptive use in rural Nepal: an event history analysis. Studies in family planning. 2011;42(2):83–92. Epub 2011/08/13. 10.1111/j.1728-4465.2011.00268.x . 21834410 PMC3338321

[39] Samandari G, Grant C, Brent L, Gullo S. "It is a thing that depends on God": barriers to delaying first birth and pursuing alternative futures among newly married adolescent girls in Niger. Reproductive health. 2019;16(1):109 Epub 2019/07/20. 10.1186/s12978-019-0757-y . 31319853 PMC6637607

