# Supplementary material for: Associations of spousal communication with contraceptive method use among adolescent wives and their husbands in Niger
Source: PLoS One. 2020 Aug 10;15(8):e0237512. doi: 10.1371/journal.pone.0237512 (PMC7416918; doi:10.1371/journal.pone.0237512)
Supplement: S1 Table — (DOCX) [file pone.0237512.s001.docx]

**S1 Table. Multivariable model of wives reports of spousal communication about contraception with all covariates**

| **Covariates** | **Spousal Communication about Contraception (Yes vs No)** |
| --- | --- |
|  | **AOR  (95% CI) p-value** |
| **Wife's Age** | **1.23**  **(1.07, 1.41)**  ***0.004*** |
| **Age Difference between Husband and Wife** | 1.02  (0.98, 1.05)  *0.35* |
| **Wife's Age at Marriage** | **0.89**  **(0.80, 0.98)**  ***0.02*** |
| **Parity** | **1.43**  **(1.16, 1.77)**  ***<0.001*** |
| **Husband's Education** |  |
| *No Education* | ref |
| *Quranic School* | 1.40  (0.87, 2.26)  *0.17* |
| *Government School* | **1.77**  **(1.18, 2.63)**  ***0.005*** |
| **Wife's Education** |  |
| *No Education* | ref |
| *Quranic School* | *1.03*  (0.64, 1.65)  *0.91* |
| *Government School* | 1.11  (0.77, 1.60)  *0.57* |
| **District** |  |
| *Loga* | ref |
| *Doutchi* | **4.17**  **(2.68, 6.48)**  ***<0.001*** |
| *Dosso* | **1.60**  **(1.05, 2.45)**  ***0.03*** |
