# Supplementary material for: Associations of spousal communication with contraceptive method use among adolescent wives and their husbands in Niger
Source: PLoS One. 2020 Aug 10;15(8):e0237512. doi: 10.1371/journal.pone.0237512 (PMC7416918; doi:10.1371/journal.pone.0237512)
Supplement: S2 Table — (DOCX) [file pone.0237512.s002.docx]

**S2 Table. Multivariable model of husbands’ reports of spousal communication about contraception with all covariates**

| **Covariates** | **Spousal Communication about Contraception (Yes vs No)** |
| --- | --- |
|  | **AOR  (95% CI) p-value** |
| **Wife's Age** | 1.00  (0.87, 1.15)  1.00 |
| **Age Difference between Husband and Wife** | 1.01  (0.98, 1.04)  *0.58* |
| **Wife's Age at Marriage** | 0.88  (0.78, 0.98)  *0.02* |
| **Parity** | **1.36**  **(1.09, 1.69)**  ***0.007*** |
| **Husband's Education** |  |
| *No Education* | ref |
| *Quranic School* | **1.70**  **(1.04, 2.76)**  ***0.03*** |
| *Government School* | **1.41**  **(0.93, 2.14)**  ***0.10*** |
| **Wife's Education** |  |
| *No Education* | ref |
| *Quranic School* | *0.72*  *(0.43, 1.19)*  *0.19* |
| *Government School* | 1.11  (0.76, 1.61)  *0.60* |
| **District** |  |
| *Loga* | ref |
| *Doutchi* | **2.66**  **(1.69, 4.20)**  ***<0.001*** |
| *Dosso* | **1.69**  **(1.10, 2.60)**  ***0.017*** |
