# Supplementary material for: Associations of spousal communication with contraceptive method use among adolescent wives and their husbands in Niger
Source: PLoS One. 2020 Aug 10;15(8):e0237512. doi: 10.1371/journal.pone.0237512 (PMC7416918; doi:10.1371/journal.pone.0237512)
Supplement: S2 File — (DOCX) [file pone.0237512.s004.docx]

| **MALE SURVEY** | | | |
| --- | --- | --- | --- |
| **Variable Name** | **Display Condition** | **Item** | **Response Options** |
|  | DEFAULT: assume that questions should appear for all unless condition is added; NOTE: =/ means does NOT equal |  |  |
| **B. HOUSEHOLD DECISION MAKING** | | | |
| 1. B_MDECISMONEY |  | Who usually decides how the money you earn will be used? | 1 RESPONDENT |
|  |  |  | 2 WIFE |
|  |  |  | 3 RESPONDENT & WIFE JOINTLY |
|  |  |  | 4 RESPONDENT'S MOTHER |
|  |  |  | 5 RESPONDENT'S FATHER |
|  |  |  | 6 RESPONDENT'S CO-WIFE |
|  |  |  | 7 SOMEONE ELSE |
|  |  |  | 997 OTHER |
|  |  |  | 998 DON'T KNOW |
|  |  |  | 999 DECLINE TO ANSWER |
| 1. B_MDECISEARN |  | Who usually decides whether or not your wife/wives should work to earn money? | 1 RESPONDENT |
|  |  |  | 2 WIFE |
|  |  |  | 3 RESPONDENT & WIFE JOINTLY |
|  |  |  | 4 RESPONDENT'S MOTHER |
|  |  |  | 5 RESPONDENT'S FATHER |
|  |  |  | 6 RESPONDENT'S CO-WIFE |
|  |  |  | 7 SOMEONE ELSE |
|  |  |  | 997 OTHER |
|  |  |  | 998 DON'T KNOW |
|  |  |  | 999 DECLINE TO ANSWER |
| 1. B_MDECISHLTH |  | Who usually makes decisions about health care for your wife/wives and children (for example, whether they can receive treatment at the CS or CSI)? | 1 RESPONDENT |
|  |  |  | 2 WIFE |
|  |  |  | 3 RESPONDENT & WIFE JOINTLY |
|  |  |  | 4 RESPONDENT'S MOTHER |
|  |  |  | 5 RESPONDENT'S FATHER |
|  |  |  | 6 RESPONDENT'S CO-WIFE |
|  |  |  | 7 SOMEONE ELSE |
|  |  |  | 997 OTHER |
|  |  |  | 998 DON'T KNOW |
|  |  |  | 999 DECLINE TO ANSWER |
| 1. B_MDECISPURCH |  | Who usually makes decisions about making major household purchases (for example, buying livestock)? | 1 RESPONDENT |
|  |  |  | 2 WIFE |
|  |  |  | 3 RESPONDENT & WIFE JOINTLY |
|  |  |  | 4 RESPONDENT'S MOTHER |
|  |  |  | 5 RESPONDENT'S FATHER |
|  |  |  | 6 RESPONDENT'S CO-WIFE |
|  |  |  | 7 SOMEONE ELSE |
|  |  |  | 997 OTHER |
|  |  |  | 998 DON'T KNOW |
|  |  |  | 999 DECLINE TO ANSWER |
| 1. B_MDECISVISIT |  | Who usually makes decisions about visits to family or relatives outside the village? | 1 RESPONDENT |
|  |  |  | 2 WIFE |
|  |  |  | 3 RESPONDENT & WIFE JOINTLY |
|  |  |  | 4 RESPONDENT'S MOTHER |
|  |  |  | 5 RESPONDENT'S FATHER |
|  |  |  | 6 RESPONDENT'S CO-WIFE |
|  |  |  | 7 SOMEONE ELSE |
|  |  |  | 997 OTHER |
|  |  |  | 998 DON'T KNOW |
|  |  |  | 999 DECLINE TO ANSWER |
| 1. B_MCHOSEMAR |  | Who had the greatest say with regard to arranging your marriage to [INDEX WIFE NAME]? | 1 RESPONDENT |
|  |  |  | 2 RESPONDENT & WIFE JOINTLY |
|  |  |  | 3 RESPONDENT WITH SOMEONE ELSE |
|  |  |  | 4 RESPONDENT'S FAMILY |
|  |  |  | 5 WIFE'S FAMILY |
|  |  |  | 6 SOMEONE ELSE |
|  |  |  | 7 JOINT DECISION NOT INCLUDING RESPONDENT |
|  |  |  | 997 OTHER |
|  |  |  | 998 DON'T KNOW |
|  |  |  | 999 DECLINE TO ANSWER |
| 1. B_MCHOSEWIF |  | Did [INDEX WIFE NAME] have a say in this decision? | 1 YES |
|  |  |  | 2 NO |
|  |  |  | 998 DON'T REMEMBER |
|  |  |  | 999 DECLINE TO ANSWER |
| 1. B_MHSHTASKA |  | How often did you do the following tasks for your family in the past 1 month: Washing clothes | 1 Never |
|  |  |  | 2 1-2 times |
|  |  |  | 3 2-15 times |
|  |  |  | 4 Daily |
|  |  |  | 998 DON'T REMEMBER |
|  |  |  | 999 DECLINE TO ANSWER |
| 1. B_MHSHTASKB |  | How often did you do the following tasks for your family in the past 1 month: Cleaning the house | 1 Never |
|  |  |  | 2 1-2 times |
|  |  |  | 3 2-15 times |
|  |  |  | Daily |
|  |  |  | 998 DON'T REMEMBER |
|  |  |  | 999 DECLINE TO ANSWER |
| 1. B_MHSHTASKC |  | How often did you do the following tasks for your family in the past 1 month: Preparing food | 1 Never |
|  |  |  | 2 1-2 times |
|  |  |  | 3 2-15 times |
|  |  |  | Daily |
|  |  |  | 998 DON'T REMEMBER |
|  |  |  | 999 DECLINE TO ANSWER |
| 1. B_MHSHDIVIS |  | Looking at this division of labor, who seems to do the most household work – you or your spouse? | 1 I DO MORE WORK |
|  |  |  | 2 MY SPOUSE DOES MORE WORK |
|  |  |  | 3 WE DO AN EQUAL AMOUNT OF WORK |
|  |  |  | 998 DON'T KNOW |
|  |  |  | 999 DECLINE TO ANSWER |
| 1. B_MCHILDCAR | if _CHILDTOT>0 | Let’s think about caring for or raising your young children. Now I will ask you whether YOU yourself perform (or performed, if in the past) a list of tasks related to that child’s care. If you have participated in caring for the child, then I will ask you how you share the task with your spouse. [INSERT SEPARATION BUT INCLUDE THIS QUESTION ON THE SAME PAGE AS INTRO] Do you participate in the daily care of the child? | 1 YES |
|  |  |  | 2 NO |
|  |  |  | 998 DON'T REMEMBER |
|  |  |  | 999 DECLINE TO ANSWER |
| 1. B_MCHILDCARA | if _CHILDTOT>0 | For each task, please indicate if it was USUALLY YOU who performed this task, if it was SHARED EQUALLY with your spouse, or it was USUALLY YOUR SPOUSE who performed this task. [INSERT BREAK BETWEEN INTRO AND QUESTION] Who typically performs the daily care of the child? | 1 USUALLY ME |
|  |  |  | 2 SHARED EQUALLY |
|  |  |  | 3 USUALLY SPOUSE |
|  |  |  | 997 OTHER |
|  |  |  | 998 DON'T REMEMBER |
|  |  |  | 999 DECLINE TO ANSWER |
| 1. B_MCHILDCARB | if _CHILDTOT>0 | Who typically plays or does various leisure time activities with the child | 1 USUALLY ME |
|  |  |  | 2 SHARED EQUALLY |
|  |  |  | 3 USUALLY SPOUSE |
|  |  |  | 997 OTHER |
|  |  |  | 998 DON'T REMEMBER |
|  |  |  | 999 DECLINE TO ANSWER |
| 1. B_MCHILDCARC | if _CHILDTOT>0 | Who typically gives the child a bath | 1 USUALLY ME |
|  |  |  | 2 SHARED EQUALLY |
|  |  |  | 3 USUALLY SPOUSE |
|  |  |  | 997 OTHER |
|  |  |  | 998 DON'T REMEMBER |
|  |  |  | 999 DECLINE TO ANSWER |
| 1. B_MCHILDCARD | if _CHILDTOT>0 | Who typically feeds the child | 1 USUALLY ME |
|  |  |  | 2 SHARED EQUALLY |
|  |  |  | 3 USUALLY SPOUSE |
|  |  |  | 997 OTHER |
|  |  |  | 998 DON'T REMEMBER |
|  |  |  | 999 DECLINE TO ANSWER |
| 1. B_MCHILDCARE | if _CHILDTOT>0 | Who typically holds the baby to sooth them when crying | 1 USUALLY ME |
|  |  |  | 2 SHARED EQUALLY |
|  |  |  | 3 USUALLY SPOUSE |
|  |  |  | 997 OTHER |
|  |  |  | 998 DON'T REMEMBER |
|  |  |  | 999 DECLINE TO ANSWER |
| 1. B_MCHILDPUNA | if _CHILDTOT>0 | Please tell me if you have used any of these methods to teach your child(ren) how to behave well or to correct them when they misbehave in the PAST MONTH. Have you spanked or slapped your child on any part of her/his body? | 1 YES |
|  |  |  | 2 NO |
|  |  |  | 998 DON'T REMEMBER |
|  |  |  | 999 DECLINE TO ANSWER |
|  |  |  | 999 DECLINE TO ANSWER |
| 1. B_MCHILDPUNB | if _CHILDTOT>0 | Please tell me if you have used any of these methods to teach your child(ren) how to behave well or to correct them when they misbehave in the PAST MONTH. Have you hit your child on the bottom or elsewhere on the body with something like a belt, stick, or other hard object | 1 YES |
|  |  |  | 2 NO |
|  |  |  | 998 DON'T REMEMBER |
|  |  |  | 999 DECLINE TO ANSWER |
| **C. PREGNANCY DESIRE** | | | |
| 1. C_MAGEFIRCH | if _IWFCH>0 | How old were you when your first child with [INDEX WIFE] was born? | [Number] Years |
| 1. C_MNUMCHILD |  | If you could choose the exact number of children to have in your whole life with [INDEX WIFE], regardless of how many children you have currently, how many would that be? | Number |
| 1. C_MIDEALBOY | If _MNUMCHILD=1-50 | How many of these children would you like to be boys? | Number |
| 1. C_MMORCHIW | If _MNUMCHILD=1-50 AND _PREG=2, 998, 999 | Would you like to have (a/another) child with [INDEX WIFE]? | 1 HAVE (ANOTHER) CHILD |
|  |  |  | 2 NO MORE/NONE |
|  |  |  | 3 SAYS THEY CAN'T GET PREGNANT |
|  |  |  | 997 OTHER |
|  |  |  | 998 UNDECIDED |
|  |  |  | 999 DECLINE TO ANSWER |
| 1. C_MWAITCHIW | If_MMORCHIW=1 | How long would you like to wait from now until [INDEX WIFE] becomes pregnant? | 1 None (want to get pregnant now) |
|  |  |  | 2 Less than a year |
|  |  |  | 3 1-2 years |
|  |  |  | 4 More than 2 years |
|  |  |  | 5 More than 5 years |
|  |  |  | 997 OTHER |
|  |  |  | 998 UNDECIDED |
|  |  |  | 999 DECLINE TO ANSWER |
| 1. C_MMORCHIWP | If _MNUMCHILD=1-50 AND _PREG=1 | IF CURRENTLY PREGNANT After the child she is expecting now, would you like to have another child with [INDEX WIFE]? | 1 HAVE ANOTHER CHILD |
|  |  |  | 2 NO MORE/NONE |
|  |  |  | 3 SAYS THEY CAN'T GET PREGNANT |
|  |  |  | 997 OTHER |
|  |  |  | 998 UNDECIDED |
|  |  |  | 999 DECLINE TO ANSWER |
| 1. C_MWAITCHIWP | If_MMORCHIWP=1 | IF YES How long would you like to wait from when [INDEX WIFE] gives birth to when she becomes pregnant again? | 1 None (want to get pregnant as soon as possible) |
|  |  |  | 2 Less than a year |
|  |  |  | 3 1-2 years |
|  |  |  | 4 More than 2 years |
|  |  |  | 5 More than 5 years |
|  |  |  | 997 OTHER |
|  |  |  | 998 UNDECIDED |
|  |  |  | 999 DECLINE TO ANSWER |
| **D. EXPOSURE TO INTERVENTION** | | | |
| 1. D_MTYPE |  | Did you know that there are things that women can do to space or delay becoming pregnant? | 1 YES |
|  |  |  | 2 No, did not know |
|  |  |  | 997 OTHER |
|  |  |  | 999 DECLINE TO ANSWER |
| 1. D_MCHW12 |  | In the last 12 months, has a community health worker visited and talked to you? | 1 YES |
|  |  |  | 2 NO |
|  |  |  | 999 DECLINE TO ANSWER |
| 1. D_MCHW12_2 | IF _MCHW12=1 | IF YES How many times? | 1 Yes, received 1 visit |
|  |  |  | 2 Yes, received 2-5 visits |
|  |  |  | 3 Yes, received more than 5 visits |
|  |  |  | 997 OTHER |
|  |  |  | 998 DON'T KNOW |
|  |  |  | 999 DECLINE TO ANSWER |
| 1. D_MCHW3 | IF _MCHW12=1 | In the last 3 months, has a community health worker visited and talked to you? | 1 YES |
|  |  |  | 2 NO |
|  |  |  | 999 DECLINE TO ANSWER |
| 1. D_MCHW3_2 | IF _MCHW3=1 | IF YES How many times? | 1 Yes, received 1 visit |
|  |  |  | 2 Yes, received 2-3 visits |
|  |  |  | 3 Yes, received more than 3 visits |
|  |  |  | 997 OTHER |
|  |  |  | 998 DON'T KNOW |
|  |  |  | 999 DECLINE TO ANSWER |
| 1. D_MCHWTOPIC | IF _MCHW12=1 | IF CHW VISITED: When the community health worker came, what topics were discussed? | 1 Topics related to FAMILY PLANNING |
|  |  |  | 2 Topics related to NUTRITION |
|  |  |  | 3 Topics related to GENDER (examples, men's responsibility to assist their wives, roles of men and women in the household, etc.) |
|  |  |  | 4 Topics related to PREGNANCY HEALTH |
|  |  |  | 5 Your wife's pregnancy at that time |
|  |  |  | 997 OTHER |
|  |  |  | 998 DON'T KNOW |
|  |  |  | 999 DECLINE TO ANSWER |
| 1. D_MCHWFP | IF _MCHW12=1 | IF CHW VISITED Did the community health worker provide you with any form of family planning? | 1 YES |
|  |  |  | 2 NO |
|  |  |  | 999 DECLINE TO ANSWER |
| 1. D_MCHWFP2 | IF _MCHWFP=1 | IF YES What type? | 1 PILL |
|  |  |  | 2 CONDOM |
|  |  |  | 3 FEMALE CONDOM |
|  |  |  | 997 OTHER |
|  |  |  | 998 DON'T KNOW |
|  |  |  | 999 DECLINE TO ANSWER |
| 1. D_MCHWACC | IF _MCHW12=1 | IF CHW VISITED Did the community health worker accompany you to the CS/CSI to help you get a form of family planning? | 1 YES |
|  |  |  | 2 NO |
|  |  |  | 999 DECLINE TO ANSWER |
| 1. D_MCHWACC2 | IF _MCHWACC=1 | IF YES What type of family planning did they take you there to get? | 1 IUD |
|  |  |  | 2 INJECTIBLES |
|  |  |  | 3 IMPLANTS |
|  |  |  | 4 PILL |
|  |  |  | 5 CONDOM |
|  |  |  | 6 FEMALE CONDOM |
|  |  |  | 7 EMERGENCY CONTRACEPTION |
|  |  |  | 997 OTHER |
|  |  |  | 998 DON'T KNOW |
|  |  |  | 999 DECLINE TO ANSWER |
| 1. D_MCHWHELP | IF _MCHW12=1 | (IF YES TO RECEIVED VISIT FROM CHW) Did you find the visit from a community health worker helpful? | 1 YES |
|  |  |  | 2 NO |
|  |  |  | 998 Not Sure |
|  |  |  | 999 DECLINE TO ANSWER |
| 1. D_MCHWYES | If _MCHWHELP=1, 998 | IF YES What was most helpful? | 1 The information was useful to help me be a better man |
|  |  |  | 2 The information helped me to speak to my wife about family planning |
|  |  |  | 3 The information helped to better understand my wife's health needs |
|  |  |  | 4 The information taught me how to better care for my wife and children |
|  |  |  | 997 OTHER |
|  |  |  | 998 DON'T KNOW |
|  |  |  | 999 DECLINE TO ANSWER |
| 1. D_MCHWYESOTH | If _MCHWYES=997 | Other_______ | Text |
| 1. D_MCHWNO | If _MCHWHELP=2 | (IF NO) Why not? | 1 I AM NOT INTERESTED IN FAMILY PLANNING |
|  |  |  | 2 I ALREADY KNEW THE INFORMATION SHE TOLD ME |
|  |  |  | 3 THE VISIT CAUSED PROBLEMS FOR ME |
|  |  |  | 997 OTHER |
|  |  |  | 998 DON'T KNOW |
|  |  |  | 999 DECLINE TO ANSWER |
| 1. D_MCHWNOOTH | if _MCHWNO=997 | Other_______ | Text |
| 1. D_MGRP12 |  | In the last 12 months, have you participated in a discussion group for men? | 1 YES |
|  |  |  | 2 NO |
|  |  |  | 999 DECLINE TO ANSWER |
| 1. D_MGRP12_2 | IF _MGRP12=1 | IF YES How many times? | 1 Participated in 1 group |
|  |  |  | 2 Yes, participated in 2-5 groups |
|  |  |  | 3 Yes, participated in more than 5 groups |
|  |  |  | 997 OTHER |
|  |  |  | 998 DON'T KNOW |
|  |  |  | 999 DECLINE TO ANSWER |
| 1. D_MGRPW3 | IF _MGRP12=1 | In the last 3 months, have you participated in a discussion group for men? | 1 YES |
|  |  |  | 2 NO |
|  |  |  | 999 DECLINE TO ANSWER |
| 1. D_MGRPW3_2 | IF _MGRPW3=1 | IF YES How many times? | 1 Participated in 1 group |
|  |  |  | 2 Yes, participated in 2-5 groups |
|  |  |  | 3 Yes, participated in more than 5 groups |
|  |  |  | 997 OTHER |
|  |  |  | 998 DON'T KNOW |
|  |  |  | 999 DECLINE TO ANSWER |
| 1. D_MGRPTOPIC | IF _MGRP12=1 | During the groups you have attended, what topics were discussed? | 1 Topics related to FAMILY PLANNING |
|  |  |  | 2 Topics related to NUTRITION |
|  |  |  | 3 Topics related to GENDER (examples, men's responsibility to assist their wives, roles of men and women in the household, etc.) |
|  |  |  | 4 Topics related to PREGNANCY HEALTH |
|  |  |  | 997 OTHER |
|  |  |  | 998 DON'T KNOW |
|  |  |  | 999 DECLINE TO ANSWER |
| 1. D_MGRPFP | IF _MGRP12=1 | During the groups, were you offered condoms? | 1 YES |
|  |  |  | 2 NO |
|  |  |  | 999 DECLINE TO ANSWER |
| 1. D_MGRPHELP | IF _MGRP12=1 | (IF YES TO PARTICIPATED IN GROUPS) Did you find the men's discussion groups helpful? | 1 YES |
|  |  |  | 2 NO |
|  |  |  | 998 Not Sure |
|  |  |  | 999 DECLINE TO ANSWER |
| 1. D_MGRPNO | If _MGRPHELP=2 | (IF NO) Why not? | 1 I AM NOT INTERESTED IN TOPICS ABOUT FAMILY PLANNING |
|  |  |  | 2 I ALREADY KNEW THE INFORMATION |
|  |  |  | 3 THE GROUP CAUSED PROBLEMS FOR ME |
|  |  |  | 997 OTHER |
|  |  |  | 998 DON'T KNOW |
|  |  |  | 999 DECLINE TO ANSWER |
| 1. D_MCHWNOOTH | if_MGRPNO=997 | Other_______ | Text |
| 1. D_MGRPYES | If _MGRPHELP=1, 998 | IF YES What was most helpful? | 1 The information was useful to help me be a better man |
|  |  |  | 2 The information helped me to speak to my wife about family planning |
|  |  |  | 3 The information helped to better understand my wife's health needs |
|  |  |  | 4 The information taught me how to better care for my wife and children |
|  |  |  | 997 OTHER |
|  |  |  | 998 DON'T KNOW |
|  |  |  | 999 DECLINE TO ANSWER |
| 1. D_MGRPYESOTH | If _MGRPYES=997 | Other_______ | Text |
| 1. D_MGRPWANT | If _MGRP12=2 | (IF NO TO PARTICIPATED IN GROUPS) Would you ever want to participate in a discussion group for men? | 1 YES |
|  |  |  | 2 NO |
|  |  |  | 998 DON'T KNOW |
|  |  |  | 999 DECLINE TO ANSWER |
| 1. D_MCSEVER |  | When was the last time you visited a health clinic for any type of health care for yourself? | 1 Never |
|  |  |  | 2 In the last month |
|  |  |  | 3 In the last year |
|  |  |  | 4 More than a year ago |
|  |  |  | 998 Don't know |
|  |  |  | 999 DECLINE TO ANSWER |
| 1. D_MCSEVERFP | IF _MCSEVER=2-4, 998 | Has any staff member during any of your visits at the health center ever spoken to you about family planning methods? | 1 YES |
|  |  |  | 2 NO |
|  |  |  | 998 DON'T REMEMBER |
|  |  |  | 999 DECLINE TO ANSWER |
| 1. D_MCS12FP | if _MCSEVER=2-3, 998 AND _MCSEVERFP=2, 998, 999 AND _MCSINITIAT=2, 998, 999 | In the last 12 months, did any staff member during any of your visits at the health center speak to you about family planning methods? | 1 YES |
|  |  |  | 2 NO |
|  |  |  | 997 OTHER |
|  |  |  | 998 DON'T REMEMBER |
|  |  |  | 999 DECLINE TO ANSWER |
|  |  |  | 999 DECLINE TO ANSWER |
| 1. D_MCSINITIAT | IF _MCSEVER=2-3, 998 | In the last 12 months, did you specifically go to a health clinic to learn about family planning methods? | 1 YES |
|  |  |  | 2 NO |
|  |  |  | 998 DON'T REMEMBER |
|  |  |  | 999 DECLINE TO ANSWER |
| 1. D_MCSFPFP | if _MCS12FP=1 OR _MCSINITIAT=1 | (IF YES) Did the health center provide or help you and your wife start using a form of family planning? | 1 No, the health center did not help us start a form of family planning |
|  |  |  | 2 IUD |
|  |  |  | 3 INJECTIBLES |
|  |  |  | 4 IMPLANTS |
|  |  |  | 5 PILL |
|  |  |  | 6 CONDOM |
|  |  |  | 7 FEMALE CONDOM |
|  |  |  | 8 EMERGENCY CONTRACEPTION |
|  |  |  | 9 LACTATION AMENAREA METHOD (LAM) |
|  |  |  | 10 OTHER TRADITIONAL METHOD |
|  |  |  | 11 ABSTINENCE |
|  |  |  | 998 DON'T KNOW |
|  |  |  | 999 DECLINE TO ANSWER |
| 1. D_MCSTYP | IF _MCSEVER=2-4, 998 | What type of health center was this? | 1 DISTRICT HOSPITAL |
|  |  |  | 2 CENTRE DE SANTÉ/CSI |
|  |  |  | 3 CASE DE SANTÉ/ACS |
|  |  |  | 4 Pharmacie |
|  |  |  | 5 Distribution à Base Communautaire/OTHER COMMUNITY HEALTH WORKER |
|  |  |  | 997 OTHER |
|  |  |  | 998 DON'T KNOW |
|  |  |  | 999 DECLINE TO ANSWER |
| 1. D_MCSANTE | if _CHILDTOT>0 | Thinking only of your most recent or youngest child, did you accompany the mother of the child to an antenatal healthcare visit? | 1 YES |
|  |  |  | 2 NO |
|  |  |  | 998 DON'T KNOW |
|  |  |  | 999 DECLINE TO ANSWER |
| 1. D_MCSANTEA | IF _MCSANTEA=1 | When you accompanied the mother of your child to antenatal visits, where did you go at the clinic? Did you: | 1 DROP HER OFF AT THE ENTRANCE OR WAIT OUTSIDE |
|  |  |  | 2 SIT IN THE WAITING ROOM |
|  |  |  | 3 JOIN HER FOR SOME OR ALL OF THE VISIT WITH THE HEALTH PROVIDER |
|  |  |  | 997 Other |
|  |  |  | 999 DECLINE TO ANSWER |
| 1. D_MCSTYPTOP |  | What type of health center does your family most typically go to when they need health care? | 1 DISTRICT HOSPITAL |
|  |  |  | 2 CENTRE DE SANTÉ/CSI |
|  |  |  | 3 CASE DE SANTÉ/ACS |
|  |  |  | 4 Pharmacie |
|  |  |  | 5 Distribution à Base Communautaire/OTHER COMMUNITY HEALTH WORKER |
|  |  |  | 6 Vendeurs Ambulants |
|  |  |  | 7 Guérisseur traditionnelle |
|  |  |  | 997 OTHER (SPECIFY) |
|  |  |  | 998 DON'T KNOW |
|  |  |  | 999 DECLINE TO ANSWER |
| 1. D_MCSHIQUAL | Input _MCSTYP to [HEALTH CENTER] in question. Make CSI default if _MCSTYP= 998, 999 | For the next set of questions I am going to read some statements to you and I want you to tell me if you agee or disagree with them. There are no right or wrong answers, we just want to know your opinions. The [HEALTH CENTER] provides high quality services | 1 AGREE |
|  |  |  | 2 DISAGREE |
|  |  |  | 998 DON'T KNOW |
|  |  |  | 999 DECLINE TO ANSWER |
| 1. D_MCSTRICK | Input _MCSTYP to [HEALTH CENTER] in question. Make CSI default if _MCSTYP= 998, 999 | Care at the [health center] is meant to trick our wives into having fewer children. | 1 AGREE |
|  |  |  | 2 DISAGREE |
|  |  |  | 998 DON'T KNOW |
|  |  |  | 999 DECLINE TO ANSWER |
| 1. D_MCSRESP | Input _MCSTYP to [HEALTH CENTER] in question. Make CSI default if _MCSTYP= 998, 999 | The staff at the [HEALTH CENTER] are friendly and respect me | 1 AGREE |
|  |  |  | 2 DISAGREE |
|  |  |  | 998 DON'T KNOW |
|  |  |  | 999 DECLINE TO ANSWER |
| 1. D_MCSALONE | Input _MCSTYP to [HEALTH CENTER] in question. Make CSI default if _MCSTYP= 998, 999 | The staff at the [HEALTH CENTER] will give my wife better service if I go with her than if she goes alone | 1 AGREE |
|  |  |  | 2 DISAGREE |
|  |  |  | 998 DON'T KNOW |
|  |  |  | 999 DECLINE TO ANSWER |
| 1. D_MCSTRUST | Input _MCSTYP to [HEALTH CENTER] in question. Make CSI default if _MCSTYP= 998, 999 | I trust that the health information my family receives at the [HEALTH CENTER] is accurate and intended to improve the health of my family | 1 AGREE |
|  |  |  | 2 DISAGREE |
|  |  |  | 998 DON'T KNOW |
|  |  |  | 999 DECLINE TO ANSWER |
| 1. D_MCSNORELIG | Input _MCSTYP to [HEALTH CENTER] in question. Make CSI default if _MCSTYP= 998, 999 | Care at the [health center] goes against Islam | 1 AGREE |
|  |  |  | 2 DISAGREE |
|  |  |  | 998 DON'T KNOW |
|  |  |  | 999 DECLINE TO ANSWER |
| 1. D_MCSINFO | Input _MCSTYP to [HEALTH CENTER] in question. Make CSI default if _MCSTYP= 998, 999 | The staff at the [HEALTH CENTER] give my wife all the information she needs for her wellbeing | 1 AGREE |
|  |  |  | 2 DISAGREE |
|  |  |  | 998 DON'T KNOW |
|  |  |  | 999 DECLINE TO ANSWER |
| 1. D_MCSMDR | Input _MCSTYP to [HEALTH CENTER] in question. Make CSI default if _MCSTYP= 998, 999 | A woman should not go to a [HEALTH CENTER] without her husband because a woman should not receive services from a male provider without her husband present | 1 AGREE |
|  |  |  | 2 DISAGREE |
|  |  |  | 998 DON'T KNOW |
|  |  |  | 999 DECLINE TO ANSWER |
| 1. D_MCSCOST | Input _MCSTYP to [HEALTH CENTER] in question. Make CSI default if _MCSTYP= 998, 999 | The cost of services at the [HEALTH CENTER] is acceptable in relation to the benefits | 1 AGREE |
|  |  |  | 2 DISAGREE |
|  |  |  | 998 DON'T KNOW |
|  |  |  | 999 DECLINE TO ANSWER |
| 1. D_MCSNOTRUST | Input _MCSTYP to [HEALTH CENTER] in question. Make CSI default if _MCSTYP= 998, 999 | Care at the [health center] cannot be trusted | 1 AGREE |
|  |  |  | 2 DISAGREE |
|  |  |  | 998 DON'T KNOW |
|  |  |  | 999 DECLINE TO ANSWER |
| 1. D_MMOBCSI |  | If she wanted to, would you allow [INDEX WIFE] to go to a health center | 1 YES |
|  |  |  | 2 NO |
|  |  |  | 998 DON'T KNOW |
|  |  |  | 999 DECLINE TO ANSWER |
| 1. D_MMOBFP | If _MMOBCSI=1, 998 | If she wanted to, would you allow [INDEX WIFE] to go to a health center to get a family planning method? | 1 YES |
|  |  |  | 2 NO |
|  |  |  | 998 DON'T KNOW |
|  |  |  | 999 DECLINE TO ANSWER |
| **E. REPRODUCTIVE HEALTH KNOWLEDGE** | | | |
| 1. E_MHLTHYTIM |  | Now I would like to talk about family planning, the ways or methods that a couple can use to delay or avoid a pregnancy. Please answer to the best of your ability. For the health of the mother and the baby, about how much time should a woman wait between giving birth and trying to become pregnant again? | 1 CORRECT (2 years/24 months) |
|  |  |  | 2 INCORRECT |
|  |  |  | 998 DON'T KNOW |
|  |  |  | 999 DECLINE TO ANSWER |
| 1. E_MHLTHYMIS |  | For the health of the mother and the baby, about how much time should a woman wait between a miscarriage (a pregnancy that did not result in a birth) and trying to become pregnant again? | 1 CORRECT (6 months) |
|  |  |  | 2 INCORRECT |
|  |  |  | 998 DON'T KNOW |
|  |  |  | 999 DECLINE TO ANSWER |
| 1. E_MPIL |  | Have you ever heard of the PILL? | 1 YES |
|  |  |  | 2 NO |
|  |  |  | 998 DON'T KNOW |
|  |  |  | 999 DECLINE TO ANSWER |
| 1. E_MPILANYW | if _MPIL=1 | Has your wife/have any of your wives used this method to space or delay pregnancy? | 1 YES |
|  |  |  | 2 NO |
|  |  |  | 998 DON'T KNOW |
|  |  |  | 999 DECLINE TO ANSWER |
| 1. E_MPILPERM | if _MPIL=1 | If your wife/one of your wives wanted to use this method to delay pregnancy, would you be okay with her doing so? | 1 YES |
|  |  |  | 2 NO |
|  |  |  | 998 DON'T KNOW |
|  |  |  | 999 DECLINE TO ANSWER |
| 1. E_MPILA | if _MPIL=1 | I am going to say a statement about this form of family planning that could be true or false. I want you to tell me if you think it is a true statement or if it is a false statement. Birth control pills are effective even if a woman misses taking them for two or three days in a row. | 1 TRUE |
|  |  |  | 2 FALSE |
|  |  |  | 998 DON'T KNOW |
|  |  |  | 999 DECLINE TO ANSWER |
| 1. E_MPILB | if _MPIL=1 | After a woman stops taking birth control pills, it's possible for her to get pregnant right away. | 1 TRUE |
|  |  |  | 2 FALSE |
|  |  |  | 998 DON'T KNOW |
|  |  |  | 999 DECLINE TO ANSWER |
| 1. E_MIUDANYW | if _MIUD=1 | Has your wife/have any of your wives used this method to space or delay pregnancy? | 1 TRUE |
|  |  |  | 2 FALSE |
|  |  |  | 998 DON'T KNOW |
|  |  |  | 999 DECLINE TO ANSWER |
| 1. E_MIUDPERM | if _MIUD=1 | If your wife/one of your wives wanted to use this method to delay pregnancy, would you be okay with her doing so? | 1 TRUE |
|  |  |  | 2 FALSE |
|  |  |  | 998 DON'T KNOW |
|  |  |  | 999 DECLINE TO ANSWER |
| 1. E_MIUD |  | Have you ever heard of an IUD? | 1 TRUE |
|  |  |  | 2 FALSE |
|  |  |  | 998 DON'T KNOW |
|  |  |  | 999 DECLINE TO ANSWER |
| 1. E_MIUDA | if _MIUD=1 | I am going to say a statement about this form of family planning that could be true or false. I want you to tell me if you think it is a true statement or if it is a false statement. An IUD cannot be felt by a woman’s husband during sex. (True) | 1 TRUE |
|  |  |  | 2 FALSE |
|  |  |  | 998 DON'T KNOW |
|  |  |  | 999 DECLINE TO ANSWER |
| 1. E_MIUDB | if _MIUD=1 | An IUD can damage a woman's inside so that she can never have children again | 1 TRUE |
|  |  |  | 2 FALSE |
|  |  |  | 998 DON'T KNOW |
|  |  |  | 999 DECLINE TO ANSWER |
| 1. E_MIUDC | if _MIUD=1 | IUDs can get permanently stuck in a woman’s body. | 1 TRUE |
|  |  |  | 2 FALSE |
|  |  |  | 998 DON'T KNOW |
|  |  |  | 999 DECLINE TO ANSWER |
| 1. E_MINJ |  | Have you ever heard of INJECTIBLES/DEPO-PROVERA? | 1 TRUE |
|  |  |  | 2 FALSE |
|  |  |  | 998 DON'T KNOW |
|  |  |  | 999 DECLINE TO ANSWER |
| 1. E_MINJANYW | if _MINJ=1 | Has your wife/have any of your wives used this method to space or delay pregnancy? | 1 TRUE |
|  |  |  | 2 FALSE |
|  |  |  | 998 DON'T KNOW |
|  |  |  | 999 DECLINE TO ANSWER |
| 1. E_MINJPERM | if _MINJ=1 | If your wife/one of your wives wanted to use this method to delay pregnancy, would you be okay with her doing so? | 1 TRUE |
|  |  |  | 2 FALSE |
|  |  |  | 998 DON'T KNOW |
|  |  |  | 999 DECLINE TO ANSWER |
| 1. E_MINJA | if _MINJ=1 | I am going to say a statement about this form of family planning that could be true or false. I want you to tell me if you think it is a true statement or if it is a false statement. Women using the birth control shot, Depo-Provera, must get an injection every three months. | 1 TRUE |
|  |  |  | 2 FALSE |
|  |  |  | 998 DON'T KNOW |
|  |  |  | 999 DECLINE TO ANSWER |
| 1. E_MINJB | if _MINJ=1 | Using Depo-Provera can cause a woman to never be able to have children again. | 1 TRUE |
|  |  |  | 2 FALSE |
|  |  |  | 998 DON'T KNOW |
|  |  |  | 999 DECLINE TO ANSWER |
| 1. E_MIMP |  | Have you ever heard of IMPLANTS? | 1 TRUE |
|  |  |  | 2 FALSE |
|  |  |  | 998 DON'T KNOW |
|  |  |  | 999 DECLINE TO ANSWER |
| 1. E_MIMPANYW | if _MIMP=1 | Has your wife/have any of your wives used this method to space or delay pregnancy? | 1 TRUE |
|  |  |  | 2 FALSE |
|  |  |  | 998 DON'T KNOW |
|  |  |  | 999 DECLINE TO ANSWER |
| 1. E_MIMPPERM | if _MIMP=1 | If your wife/one of your wives wanted to use this method to delay pregnancy, would you be okay with her doing so? | 1 TRUE |
|  |  |  | 2 FALSE |
|  |  |  | 998 DON'T KNOW |
|  |  |  | 999 DECLINE TO ANSWER |
| 1. E_MIMPA | if _MIMP=1 | I am going to say a statement about this form of family planning that could be true or false. I want you to tell me if you think it is a true statement or if it is a false statement. Long-acting methods like the implant or IUD can be removed early if a woman changes her mind about wanting to get pregnant. | 1 TRUE |
|  |  |  | 2 FALSE |
|  |  |  | 998 DON'T KNOW |
|  |  |  | 999 DECLINE TO ANSWER |
| 1. E_MCON |  | Have you ever heard of the MALE CONDOMS? | 1 TRUE |
|  |  |  | 2 FALSE |
|  |  |  | 998 DON'T KNOW |
|  |  |  | 999 DECLINE TO ANSWER |
| 1. E_MCONANYW | if _MCON=1 | Have you ever used this method to space or delay pregnancy? | 1 TRUE |
|  |  |  | 2 FALSE |
|  |  |  | 998 DON'T KNOW |
|  |  |  | 999 DECLINE TO ANSWER |
| 1. E_MCONPERM | if _MCON=1 | If your wife/one of your wives wanted you to use this method to delay pregnancy, would you be willing to do so? | 1 TRUE |
|  |  |  | 2 FALSE |
|  |  |  | 998 DON'T KNOW |
|  |  |  | 999 DECLINE TO ANSWER |
| 1. E_MCONA | if _MCON=1 | I am going to say a statement about this form of family planning that could be true or false. I want you to tell me if you think it is a true statement or if it is a false statement. It is okay to use the same condom more than once. | 1 TRUE |
|  |  |  | 2 FALSE |
|  |  |  | 998 DON'T KNOW |
|  |  |  | 999 DECLINE TO ANSWER |
| 1. E_MLAM |  | Have you ever heard of LACTATIONAL AMENORRHEA METHOD (LAM)? | 1 TRUE |
|  |  |  | 2 FALSE |
|  |  |  | 998 DON'T KNOW |
|  |  |  | 999 DECLINE TO ANSWER |
| 1. E_MLAMANYW | if _MLAM=1 | Has your wife/have any of your wives used this method to space or delay pregnancy? | 1 TRUE |
|  |  |  | 2 FALSE |
|  |  |  | 998 DON'T KNOW |
|  |  |  | 999 DECLINE TO ANSWER |
| 1. E_MLAMPERM | if _MLAM=1 | If your wife/one of your wives wanted to use this method to delay pregnancy, would you be okay with her doing so? | 1 TRUE |
|  |  |  | 2 FALSE |
|  |  |  | 998 DON'T KNOW |
|  |  |  | 999 DECLINE TO ANSWER |
| 1. E_MLAMA | if _MLAM=1 | Breastfeeding is an effective form of avoiding pregnancy even after the woman's menstrual bleeding has returned since giving birth (False) | 1 TRUE |
|  |  |  | 2 FALSE |
|  |  |  | 998 DON'T KNOW |
|  |  |  | 999 DECLINE TO ANSWER |
| 1. E_MLAMB | if _MLAM=1 | Exclusive breastfeeding means the woman breastfeeds her baby “on demand,” day and night, and does not give any other food, water or liquid (True) | 1 TRUE |
|  |  |  | 2 FALSE |
|  |  |  | 998 DON'T KNOW |
|  |  |  | 999 DECLINE TO ANSWER |
| 1. E_MKWLMENARC |  | Generally speaking, once a female gets her first menstrual period, she can get pregnant | 1 TRUE |
|  |  |  | 2 FALSE |
|  |  |  | 998 DON'T KNOW |
|  |  |  | 999 DECLINE TO ANSWER |
| 1. E_MKWLFERT |  | From one menstrual period to the next, are there certain days when a woman is more likely to become pregnant? | 1 TRUE |
|  |  |  | 2 FALSE |
|  |  |  | 998 DON'T KNOW |
|  |  |  | 999 DECLINE TO ANSWER |
| 1. E_MKWLFERTA | if _MKWLFERT=1 | Is this time just before her period begins, during her period, right after her period has ended, or halfway between two periods? | 1 JUST BEFORE HER PERIOD BEGINS |
|  |  |  | 2 DURING HER PERIOD |
|  |  |  | 3 RIGHT AFTER HER PERIOD HAS ENDED |
|  |  |  | 4 HALFWAY BETWEEN TWO PERIODS |
|  |  |  | 997 OTHER |
|  |  |  | 998 DON'T KNOW |
|  |  |  | 999 DECLINE TO ANSWER |
| 1. E_MKWLFERTPOST |  | After the birth of a child, can a woman become pregnant before her menstrual period has returned? | 1 TRUE |
|  |  |  | 2 FALSE |
|  |  |  | 998 DON'T KNOW |
|  |  |  | 999 DECLINE TO ANSWER |
| 1. E_MFPPLACE |  | Do you know of a place where you and your wife could obtain family planning assistance if you wished to start using a method of family planning? | 1 TRUE |
|  |  |  | 2 FALSE |
|  |  |  | 998 DON'T KNOW |
|  |  |  | 999 DECLINE TO ANSWER |
| 1. E_MFPWHERE | if _MFPPLACE=1 | Where is that? | 1 DISTRICT HOSPITAL |
|  |  |  | 2 CENTRE DE SANTÉ/CSI |
|  |  |  | 3 CASE DE SANTÉ/ACS |
|  |  |  | 4 Pharmacie |
|  |  |  | 5 Distribution à Base Communautaire/OTHER COMMUNITY HEALTH WORKER |
|  |  |  | 6 Vendeurs Ambulants |
|  |  |  | 7 Guérisseur traditionnelle |
|  |  |  | 8 FRIEND/RELATIVE |
|  |  |  | 997 OTHER (SPECIFY) |
|  |  |  | 998 DON'T KNOW |
|  |  |  | 999 DECLINE TO ANSWER |
| 1. E_MFPWHEREOTH | if_MFPWHERE=997 | Other_______ | Text |
| 1. E_MHEINFOWHO |  | What person in your life do you trust most when it comes to getting information on spacing or delaying pregnancy? | 1 FATHER |
|  |  |  | 2 FRIEND |
|  |  |  | 3 BROTHER |
|  |  |  | 4 ASC from the CS |
|  |  |  | 5 OTHER COMMUNITY HEALTH WORKER |
|  |  |  | 6 NURSE/DOCTOR AT CSI |
|  |  |  | 7 MOTHER |
|  |  |  | 8 VILLAGE LEADER |
|  |  |  | 9 OTHER RELATIVE |
|  |  |  | 10 NO ONE |
|  |  |  | 997 OTHER |
|  |  |  | 998 DON'T KNOW |
|  |  |  | 999 DECLINE TO ANSWER |
| **G. CONTRACEPTION DECISIONS** | | | |
|  |  | Now, I’m going to read you some decisions that might be made in a family, and then list some family members: I will show you this picture of a ladder – imagine those at the top of the ladder have the most influence in the family over a decision and those in the bottom have no influence over that decision. For each type of decision I read to you I would like you tell me how much influence each person has for that decision. | NOT A QUESTION |
| 1. G_MDECISCHILD |  | How much influence does this person have over the decision of how many children you should have? | Husband |
|  |  |  | Mother |
|  |  |  | Wife |
|  |  |  | Husband's Brother |
|  |  |  | Father |
|  |  |  | Co-wife |
| 1. G_MDECISCHILDTOP |  | Who has the most influence in the family over the decision on how many children you should have? | Husband |
|  |  |  | Mother |
|  |  |  | Wife |
|  |  |  | Husband's Brother |
|  |  |  | Father |
|  |  |  | Co-wife |
| 1. G_MDECISFP |  | Now, I’m going to read you some decisions that might be made in a family, and then list some family members: I will show you this picture of a ladder – imagine those at the top of the ladder have the most influence in the family over a decision and those in the bottom have no influence over that decision. For each type of decision I read to you I would like you tell me how much influence each person has for that decision. How much influence does this person have on the decision on whether you or [INDEX WIFE] should use a family planning method to space births? | Husband |
|  |  |  | Mother |
|  |  |  | Wife |
|  |  |  | Husband's Brother |
|  |  |  | Father |
|  |  |  | Co-wife |
| 1. G_MDECISFPTOP |  | Who has the most influence in the family over the decision on whether you or [INDEX WIFE] should use a family planning method to space births? | Husband |
|  |  |  | Mother |
|  |  |  | Wife |
|  |  |  | Husband's Brother |
|  |  |  | Father |
|  |  |  | Co-wife |
| **H. CONTRACEPTION BELIEFS** | | | |
| 1. H_MFPNOFP |  | What are some reasons why a husband would not allow his wife to use a family planning method to delay pregnancy? (CHECK ALL THAT APPLY) | 1 HE WANTS CHILDREN |
|  |  |  | 2 IT IS AGAINST RELIGION BELIEFS |
|  |  |  | 3 IT IS BAD FOR HEALTH TO DELAY PREGNANCY |
|  |  |  | 4 OTHER PEOPLE WOULD NOT BE ACCEPTING |
|  |  |  | 5 ANOTHER MAN MIGHT TAKE ADVANTAGE OF HER SEXUALLY |
|  |  |  | 6 I do not trust the government or outsiders who are providing family planning |
|  |  |  | 7 Someone would need to touch my wife's body for her to get family planning |
|  |  |  | 997 OTHER |
|  |  |  | 998 DON'T KNOW |
|  |  |  | 999 DECLINE TO ANSWER |
| 1. H_MFPNOFPM |  | What is the main reason why a husband would not allow his wife to use a family planning method to delay pregnancy? | 1 HE WANTS CHILDREN |
|  |  |  | 2 IT IS AGAINST RELIGION BELIEFS |
|  |  |  | 3 IT IS BAD FOR HEALTH TO DELAY PREGNANCY |
|  |  |  | 4 OTHER PEOPLE WOULD NOT BE ACCEPTING |
|  |  |  | 5 ANOTHER MAN MIGHT TAKE ADVANTAGE OF HER SEXUALLY |
|  |  |  | 6 I do not trust the government or outsiders who are providing family planning |
|  |  |  | 7 Someone would need to touch my wife's body for her to get family planning |
|  |  |  | 997 OTHER |
|  |  |  | 998 DON'T KNOW |
|  |  |  | 999 DECLINE TO ANSWER |
| 1. H_MMARAGE |  | How long after a girl is married should she become pregnant? | 1 As soon as possible |
|  |  |  | 2 Within the first 6 months |
|  |  |  | 3 6 months to 1 year |
|  |  |  | 4 1-2 years |
|  |  |  | 5 2 years or more |
|  |  |  | 997 OTHER |
|  |  |  | 998 DON'T KNOW |
|  |  |  | 999 DECLINE TO ANSWER |
| 1. H_MBELHIMP |  | For the next set of questions I am going to read some statements to you and I want you to tell me if you agee or disagree with them. There are no right or wrong answers, we just want to know your opinions. I feel it is important to be sure my wife and our children are healthy by waiting a healthy amount of time in between pregnancies | 1 AGREE |
|  |  |  | 2 DISAGREE |
|  |  |  | 998 DON'T KNOW |
|  |  |  | 999 DECLINE TO ANSWER |
| 1. H_MBELHELP |  | I would help my wife if she wanted to wait two years after giving birth to a child to get pregnant again | 1 AGREE |
|  |  |  | 2 DISAGREE |
|  |  |  | 998 DON'T KNOW |
|  |  |  | 999 DECLINE TO ANSWER |
| 1. H_MBELISLAMCH |  | Islam dictates that we must have as many children as God allows | 1 AGREE |
|  |  |  | 2 DISAGREE |
|  |  |  | 998 DON'T KNOW |
|  |  |  | 999 DECLINE TO ANSWER |
| 1. H_MBELDIE |  | I believe that using a contraceptive method could help reduce the risk of my wife or a new child dying | 1 AGREE |
|  |  |  | 2 DISAGREE |
|  |  |  | 998 DON'T KNOW |
|  |  |  | 999 DECLINE TO ANSWER |
| 1. H_MBELHUSRES |  | It is man's responsibility to make sure his wife will not get pregnant if it’s too soon since her last child was born | 1 AGREE |
|  |  |  | 2 DISAGREE |
|  |  |  | 998 DON'T KNOW |
|  |  |  | 999 DECLINE TO ANSWER |
| 1. H_MBELBOTHRES |  | It is the responsibility of both the woman and her husband to avoid pregnancy | 1 AGREE |
|  |  |  | 2 DISAGREE |
|  |  |  | 998 DON'T KNOW |
|  |  |  | 999 DECLINE TO ANSWER |
| 1. H_MBELFPSX |  | Having sex is not as enjoyable if the woman is using a family planning method to delay pregnancy | 1 AGREE |
|  |  |  | 2 DISAGREE |
|  |  |  | 998 DON'T KNOW |
|  |  |  | 999 DECLINE TO ANSWER |
| 1. H_MBELMDECFP |  | Only the man should decide whether to use a family planning method to delay pregnancy | 1 AGREE |
|  |  |  | 2 DISAGREE |
|  |  |  | 998 DON'T KNOW |
|  |  |  | 999 DECLINE TO ANSWER |
| 1. H_MBELISLAMNO |  | Islam does not approve of the use of family planning | 1 AGREE |
|  |  |  | 2 DISAGREE |
|  |  |  | 998 DON'T KNOW |
|  |  |  | 999 DECLINE TO ANSWER |
| 1. H_MBELMDECCH |  | It is the husband who should decide how many children to have. | 1 AGREE |
|  |  |  | 2 DISAGREE |
|  |  |  | 998 DON'T KNOW |
|  |  |  | 999 DECLINE TO ANSWER |
| 1. H_MBELCODECCH |  | A couple should decide together how many children to have. | 1 AGREE |
|  |  |  | 2 DISAGREE |
|  |  |  | 998 DON'T KNOW |
|  |  |  | 999 DECLINE TO ANSWER |
| 1. H_MBELWDECFP |  | The woman has the right to decide to use contraceptives even if her husband does not agree | 1 AGREE |
|  |  |  | 2 DISAGREE |
|  |  |  | 998 DON'T KNOW |
|  |  |  | 999 DECLINE TO ANSWER |
| 1. H_MBELFPHEALTH |  | Using a family planning method will not help reduce health problems for my wives or future children | 1 AGREE |
|  |  |  | 2 DISAGREE |
|  |  |  | 998 DON'T KNOW |
|  |  |  | 999 DECLINE TO ANSWER |
| 1. H_MBELCODECFP |  | A man and a woman should decide together what contraceptive method they will use. | 1 AGREE |
|  |  |  | 2 DISAGREE |
|  |  |  | 998 DON'T KNOW |
|  |  |  | 999 DECLINE TO ANSWER |
| 1. H_MBELWIDEC |  | The woman can decide what type of contraceptive to use because she is the one who will use it. | 1 AGREE |
|  |  |  | 2 DISAGREE |
|  |  |  | 998 DON'T KNOW |
|  |  |  | 999 DECLINE TO ANSWER |
| 1. H_MBELRELIGYES |  | My religion allows the healthy spacing of births | 1 AGREE |
|  |  |  | 2 DISAGREE |
|  |  |  | 998 DON'T KNOW |
|  |  |  | 999 DECLINE TO ANSWER |
| 1. H_MBELALLOWFP |  | A real man does not allow his wives to use family planning | 1 AGREE |
|  |  |  | 2 DISAGREE |
|  |  |  | 998 DON'T KNOW |
|  |  |  | 999 DECLINE TO ANSWER |
| 1. H_MBELTRICK |  | Family planning and those that promote it are being used by the governemnt to trick us into having fewer children | 1 AGREE |
|  |  |  | 2 DISAGREE |
|  |  |  | 998 DON'T KNOW |
|  |  |  | 999 DECLINE TO ANSWER |
| **F. CONTRACEPTION USE** | | | |
| 1. F_MFPEVER |  | Now I would like to ask about you and your wife's use of family planning. Have you or [INDEX WIFE] ever done something or used any method to space or delay getting pregnant? | 1 YES |
|  |  |  | 2 NO |
|  |  |  | 999 DECLINE TO ANSWER |
| 1. F_MFPCURUSE | if _MFPEVER=1 | Are you or [INDEX WIFE] currently doing something or using any method to delay or avoid getting pregnant? | 1 YES |
|  |  |  | 2 NO |
|  |  |  | 999 DECLINE TO ANSWER |
| 1. F_MFPCURTYP | if _MFPCURUSE=1 | Which method are you or [INDEX WIFE] currently using? | 1 No method |
|  |  |  | 2 IUD |
|  |  |  | 3 INJECTIBLES |
|  |  |  | 4 IMPLANTS |
|  |  |  | 5 PILL |
|  |  |  | 6 CONDOM |
|  |  |  | 7 FEMALE CONDOM |
|  |  |  | 8 EMERGENCY CONTRACEPTION |
|  |  |  | 9 LACTATION AMENAREA METHOD (LAM) |
|  |  |  | 10 OTHER TRADITIONAL METHOD |
|  |  |  | 11 ABSTINENCE |
|  |  |  | 998 DON'T KNOW |
|  |  |  | 999 DECLINE TO ANSWER |
| 1. F_MFPCURTYPTOT | if _MFPCURTYP=2-11 | To confirm, in total, you are currently using [TOTAL NUMBER OF CURRENT METHODS] types of family planning? | 1 CORRECT |
|  |  |  | 2 INCORRECT |
|  |  |  | 999 DECLINE TO ANSWER |
| 1. F_MFPCURLAM | if _MFPCURTYP=9 | (IF LAM) Do you give the baby anything to eat or drink besides breast milk? | 1 YES |
|  |  |  | 2 NO |
|  |  |  | 998 DON'T KNOW |
| 1. F_MFPCURSTART | if _MFPCURTYP=2-11 | How long ago did you/she first start using (CURRENT METHOD)? | Number |
| 1. F_MFPCURTIM | if _MFPCURTYP=2-11 | For how long have you been using (CURRENT METHOD) now without stopping? | Number |
| 1. F_MFPPRIOR | if _MFPEVER=1 | Prior to what you are doing now, have you or [INDEX WIFE] done something or used any method to delay or avoid getting pregnant in the past? | 1 YES |
|  |  |  | 2 NO |
|  |  |  | 999 DECLINE TO ANSWER |
| 1. F_MFPPRIORTYPA | if _MFPPRIOR=1 | IF YES Which method or methods have you and [INDEX WIFE] used? | 1 No method |
|  |  |  | 2 IUD |
|  |  |  | 3 INJECTIBLES |
|  |  |  | 4 IMPLANTS |
|  |  |  | 5 PILL |
|  |  |  | 6 CONDOM |
|  |  |  | 7 FEMALE CONDOM |
|  |  |  | 8 EMERGENCY CONTRACEPTION |
|  |  |  | 9 LACTATION AMENAREA METHOD (LAM) |
|  |  |  | 10 OTHER TRADITIONAL METHOD |
|  |  |  | 11 ABSTINENCE |
|  |  |  | 998 DON'T KNOW |
|  |  |  | 999 DECLINE TO ANSWER |
| 1. F_MFPTOTUSE | if _MFPCURTYPA=2-11 | To confirm, in total you and [INDEX WIFE] have used _____(TOTAL) methods to delay or avoid getting pregnant prior to what you are using currently. Is that correct? | 1 YES |
|  |  |  | 2 NO |
|  |  |  | 999 DECLINE TO ANSWER |
| 1. F_MFPPRIORTIMA | Display question series (_MFPPRIORTIMA, _FPPRIORSTOPA) once for every method (x=total in _MFPTOTUSE) | For how long did you use (METHOD)? | Number |
| 1. F_MFPPRIORSTOPA |  | Why did you stop using (METHOD)? | 1 NOT HAVING SEX |
|  |  |  | 2 INFREQUENT SEX |
|  |  |  | 3 WIFE CAN'T GET PREGNANT |
|  |  |  | 4 WIFE IS BREASTFEEDING |
|  |  |  | 5 UP TO GOD/FATALISTIC |
|  |  |  | 6 RESPONDENT OPPOSED |
|  |  |  | 7 WIFE OPPOSED |
|  |  |  | 8 OTHERS OPPOSED |
|  |  |  | 9 RELIGIOUS PROHIBITION |
|  |  |  | 10 KNOWS NO METHOD |
|  |  |  | 11 KNOWS NO SOURCE |
|  |  |  | 12 SIDE EFFECTS/HEALTH CONCERNS |
|  |  |  | 13 LACK OF ACCESS/TOO FAR |
|  |  |  | 14 COSTS TOO MUCH |
|  |  |  | 15 PREFERRED METHOD NOT AVAILABLE |
|  |  |  | 16 NO METHOD AVAILABLE |
|  |  |  | 17 INCONVENIENT TO USE |
|  |  |  | 18 INTERFERES WITH BODY'S NORMAL PROCESSES |
|  |  |  | 19 Want children |
|  |  |  | 20 Became pregnant |
|  |  |  | 997 OTHER |
|  |  |  | 998 DON'T KNOW |
|  |  |  | 999 DECLINE TO ANSWER |
| 1. F_MFPLSTSEX | if _MFPEVER=1 AND _PREG=2, 998, 999 | Did you use a method of family planning the last time that you had sex with [INDEX WIFE]? | 1 No method |
|  |  |  | 2 IUD |
|  |  |  | 3 INJECTIBLES |
|  |  |  | 4 IMPLANTS |
|  |  |  | 5 PILL |
|  |  |  | 6 CONDOM |
|  |  |  | 7 FEMALE CONDOM |
|  |  |  | 8 EMERGENCY CONTRACEPTION |
|  |  |  | 9 LACTATION AMENAREA METHOD (LAM) |
|  |  |  | 10 OTHER TRADITIONAL METHOD |
|  |  |  | 11 ABSTINENCE |
|  |  |  | 998 DON'T KNOW |
|  |  |  | 999 DECLINE TO ANSWER |
| 1. F_MFPNOUSE | if _MFPEVER=2, 999 or _FPCURUSE=2, 999 or _FPPRIORA=2, 999 | (IF NOT USING) What are your reasons for not using a family planning method to delay pregnancy | 1 NOT HAVING SEX |
|  |  |  | 2 INFREQUENT SEX |
|  |  |  | 3 WIFE CAN'T GET PREGNANT |
|  |  |  | 4 WIFE IS BREASTFEEDING |
|  |  |  | 5 UP TO GOD/FATALISTIC |
|  |  |  | 6 RESPONDENT OPPOSED |
|  |  |  | 7 WIFE OPPOSED |
|  |  |  | 8 OTHERS OPPOSED |
|  |  |  | 9 RELIGIOUS PROHIBITION |
|  |  |  | 10 KNOWS NO METHOD |
|  |  |  | 11 KNOWS NO SOURCE |
|  |  |  | 12 SIDE EFFECTS/HEALTH CONCERNS |
|  |  |  | 13 LACK OF ACCESS/TOO FAR |
|  |  |  | 14 COSTS TOO MUCH |
|  |  |  | 15 PREFERRED METHOD NOT AVAILABLE |
|  |  |  | 16 NO METHOD AVAILABLE |
|  |  |  | 17 INCONVENIENT TO USE |
|  |  |  | 18 INTERFERES WITH BODY'S NORMAL PROCESSES |
|  |  |  | 19 Want children |
|  |  |  | 20 Became pregnant |
|  |  |  | 997 OTHER |
|  |  |  | 998 DON'T KNOW |
|  |  |  | 999 DECLINE TO ANSWER |
| 1. F_MFPFIRST | If _MFPEVER=1 | The first time you used a family planning method to delay pregnancy with [INDEX WIFE], how many living children did you have at that time, if any? | Number |
| 1. F_MFPN3B | if _MFPCURUSE=1 | (IF CURRENTLY USING) Will you continue to use (CURRENT METHOD) with [INDEX WIFE] over the next 3 months to space or delay pregnancy? | 1 YES |
|  |  |  | 2 NO |
|  |  |  | 998 DON'T KNOW |
|  |  |  | 999 DECLINE TO ANSWER |
| 1. F_MFPN3B2 | if _MFPCURTYPTOT>1 AND _MFPN3B=1 | Which of those methods will you continue to use over the next 3 months? | 1 No method |
|  |  |  | 2 IUD |
|  |  |  | 3 INJECTIBLES |
|  |  |  | 4 IMPLANTS |
|  |  |  | 5 PILL |
|  |  |  | 6 CONDOM |
|  |  |  | 7 FEMALE CONDOM |
|  |  |  | 8 EMERGENCY CONTRACEPTION |
|  |  |  | 9 LACTATION AMENAREA METHOD (LAM) |
|  |  |  | 10 OTHER TRADITIONAL METHOD |
|  |  |  | 11 ABSTINENCE |
|  |  |  | 998 DON'T KNOW |
|  |  |  | 999 DECLINE TO ANSWER |
| 1. F_MFPN3A | if _MFPN3B=2 OR if _MFPCURUSE=2, 999 | (IF NOT CURRENTLY USING) Will you use a family planning method with [INDEX WIFE] in the next 3 months to avoid or delay pregnancy? | 1 YES |
|  |  |  | 2 NO |
|  |  |  | 998 DON'T KNOW |
|  |  |  | 999 DECLINE TO ANSWER |
| 1. F_MFPNPOSTP | if _PREG=1 | After your current pregnancy is over, will you use a family planning method to space or delay pregnancy? | 1 YES |
|  |  |  | 2 NO |
|  |  |  | 998 DON'T KNOW |
|  |  |  | 999 DECLINE TO ANSWER |
| 1. F_MFPN3TYP | If _MFPN3A=1 OR _MFPNPOSTP=1 | Which contraceptive method would you prefer to use with her? | 1 No method |
|  |  |  | 2 IUD |
|  |  |  | 3 INJECTIBLES |
|  |  |  | 4 IMPLANTS |
|  |  |  | 5 PILL |
|  |  |  | 6 CONDOM |
|  |  |  | 7 FEMALE CONDOM |
|  |  |  | 8 EMERGENCY CONTRACEPTION |
|  |  |  | 9 LACTATION AMENAREA METHOD (LAM) |
|  |  |  | 10 OTHER TRADITIONAL METHOD |
|  |  |  | 11 ABSTINENCE |
|  |  |  | 998 DON'T KNOW |
|  |  |  | 999 DECLINE TO ANSWER |
| **I. FAMILY PLANNING COMMUNICATION** | | | |
| 1. I_MCONVW | if _POLYTOT>1 | Have you ever had a conversation with any of your wives about doing something to space or delay pregnancy? | 1 YES |
|  |  |  | 2 NO |
|  |  |  | 998 DON'T REMEMBER |
|  |  |  | 999 DECLINE TO ANSWER |
| 1. I_MIWCONVIW |  | Have you ever had a conversation with [INDEX WIFE] about doing something to space or delay pregnancy? | 1 YES |
|  |  |  | 2 NO |
|  |  |  | 998 DON'T REMEMBER |
|  |  |  | 999 DECLINE TO ANSWER |
| 1. I_MIWTMCONV12 | if _MIWTMCONV=1 | Did you have a discussion in the past twelve months with [INDEX WIFE] about how much time you will wait between births? | 1 YES |
|  |  |  | 2 NO |
|  |  |  | 998 DON'T REMEMBER |
|  |  |  | 999 DECLINE TO ANSWER |
| 1. I_MIWTMTELL | if _MIWTMCONV=1 | (IF YES TO EVER CONVERSATION) During that conversation, did she tell you her wishes about how much time she would like to wait between births? | 1 YES, she told you she wanted to wait 2 YEARS OR MORE in between births |
|  |  |  | 2 YES, she told you she wanted to wait FEWER THAN 2 YEARS in between births |
|  |  |  | 3 YES, she told you she does not want to wait or does not care if she waits in between births |
|  |  |  | 4 NO, she did not share her wishes |
|  |  |  | 997 OTHER |
|  |  |  | 998 DON'T REMEMBER |
|  |  |  | 999 DECLINE TO ANSWER |
| 1. I_MIWFPCONV | if _MIWTMCONV=1 | Did you have a discussion with [INDEX WIFE] in the past twelve months about using a method of family planning to space births? | 1 YES |
|  |  |  | 2 NO |
|  |  |  | 998 DON'T REMEMBER |
|  |  |  | 999 DECLINE TO ANSWER |
| 1. I_MIWFPTELL | if _MIWFPCONV=1 | (IF YES TO EVER CONVERSATION) During that conversation, did she tell you her wishes about her using a method of family planning to space births? | 1 YES, she told you she wanted to use a family planning method |
|  |  |  | 2 YES, she told you she did not want to use a family planning method |
|  |  |  | 3 NO, she did not share her wishes |
|  |  |  | 997 OTHER |
|  |  |  | 998 DON'T REMEMBER |
|  |  |  | 999 DECLINE TO ANSWER |
| 1. I_MIWFPYES | if _MIWTMCONV=1 OR if _MIWFPCONV=1 | After you two spoke, did you decide that you would like her to use a family planning method to delay pregnancy ? | 1 YES |
|  |  |  | 2 NO |
|  |  |  | 998 DON'T REMEMBER |
|  |  |  | 999 DECLINE TO ANSWER |
| 1. I_MOTHCONV |  | Have you ever had a conversation with (other) family members or friends about things people do to delay pregnancy? | 1 YES |
|  |  |  | 2 NO |
|  |  |  | 998 DON'T REMEMBER |
|  |  |  | 999 DECLINE TO ANSWER |
| 1. I_MOTHCONVW | If _MOTHCONV=1 | IF YES, with whom have you had a conversation with about delaying pregnancy? | 1 SON |
|  |  |  | 2 FRIEND |
|  |  |  | 3 FATHER |
|  |  |  | 4 MOTHER |
|  |  |  | 5 BROTHER |
|  |  |  | 6 SISTER |
|  |  |  | 7 MALE COUSIN |
|  |  |  | 8 FEMALE COUSIN |
|  |  |  | 997 OTHER |
|  |  |  | 998 DON'T REMEMBER |
|  |  |  | 999 DECLINE TO ANSWER |
| 1. I_MOTHCONVWH | If _MOTHCONV=1 | IF YES, what did you say about delaying pregnancy during such conversation(s)? | 1 I recommended delaying pregnancy |
|  |  |  | 2 I am considering doing something to delay pregnancy |
|  |  |  | 3 I would never do something to delay pregnancy |
|  |  |  | 4 I disapprove of intentionally delaying pregnancy |
|  |  |  | 5 I do not have a strong opinion about delaying pregnancy |
|  |  |  | 997 OTHER (Specify) |
|  |  |  | 998 DON'T REMEMBER |
|  |  |  | 999 DECLINE TO ANSWER |
| 1. I.MOTHCONVWHOTH | if_MOTHCONVWH=997 | Other_______ | Text |
| **J. LIFE SATISFACTION** | | | |
| 1. J_MDEPRESSA |  | [INTRO] Now I would like to ask you some questions about how you have been feeling. I would like to know if in the last 7 days (or one week) how often have you been bothered by each of the following. [SEPARATION] In the last 7 days have you: Felt down, depressed, irritable, or hopeless? | 1 Not at all |
|  |  |  | 2 Some days |
|  |  |  | 3 Nearly every day |
|  |  |  | 997 OTHER |
|  |  |  | 999 DECLINE TO ANSWER |
| 1. J_MDEPRESSB |  | [INTRO] Now I would like to ask you some questions about how you have been feeling. I would like to know if in the last 7 days (or one week) how often have you been bothered by each of the following. [SEPARATION] In the last 7 days have you: Felt little interest or pleasure in doing things? | 1 Not at all |
|  |  |  | 2 Some days |
|  |  |  | 3 Nearly every day |
|  |  |  | 997 OTHER |
|  |  |  | 999 DECLINE TO ANSWER |
| 1. J_MDEPRESSC |  | [INTRO] Now I would like to ask you some questions about how you have been feeling. I would like to know if in the last 7 days (or one week) how often have you been bothered by each of the following. [SEPARATION] In the last 7 days have you: Had trouble falling asleep, staying asleep, or sleeping too much? | 1 Not at all |
|  |  |  | 2 Some days |
|  |  |  | 3 Nearly every day |
|  |  |  | 997 OTHER |
|  |  |  | 999 DECLINE TO ANSWER |
| 1. J_MDEPRESSD |  | [INTRO] Now I would like to ask you some questions about how you have been feeling. I would like to know if in the last 7 days (or one week) how often have you been bothered by each of the following. [SEPARATION] In the last 7 days have you: Had a poor appetite, weight loss, or overeating? | 1 Not at all |
|  |  |  | 2 Some days |
|  |  |  | 3 Nearly every day |
|  |  |  | 997 OTHER |
|  |  |  | 999 DECLINE TO ANSWER |
| 1. J_MDEPRESSE |  | [INTRO] Now I would like to ask you some questions about how you have been feeling. I would like to know if in the last 7 days (or one week) how often have you been bothered by each of the following. [SEPARATION] In the last 7 days have you: Felt tired, or like you have little energy? | 1 Not at all |
|  |  |  | 2 Some days |
|  |  |  | 3 Nearly every day |
|  |  |  | 997 OTHER |
|  |  |  | 999 DECLINE TO ANSWER |
| 1. J_MDEPRESSF |  | [INTRO] Now I would like to ask you some questions about how you have been feeling. I would like to know if in the last 7 days (or one week) how often have you been bothered by each of the following. [SEPARATION] In the last 7 days have you: Felt bad about yourself—or felt that you are a failure, or that you have let yourself or your family down? | 1 Not at all |
|  |  |  | 2 Some days |
|  |  |  | 3 Nearly every day |
|  |  |  | 997 OTHER |
|  |  |  | 999 DECLINE TO ANSWER |
| 1. J_MDEPRESSG |  | [INTRO] Now I would like to ask you some questions about how you have been feeling. I would like to know if in the last 7 days (or one week) how often have you been bothered by each of the following. [SEPARATION] In the last 7 days have you: Had thoughts that you would be better off dead, or of hurting yourself in some way? | 1 Not at all |
|  |  |  | 2 Some days |
|  |  |  | 3 Nearly every day |
|  |  |  | 997 OTHER |
|  |  |  | 999 DECLINE TO ANSWER |
| 1. J_MHOPEGD |  | I expect good things to happen to me. | 1 AGREE |
|  |  |  | 2 DISAGREE |
|  |  |  | 998 DON'T KNOW |
|  |  |  | 999 DECLINE TO ANSWER |
| 1. J_MHOPEEXC |  | I am excited about my future. | 1 AGREE |
|  |  |  | 2 DISAGREE |
|  |  |  | 998 DON'T KNOW |
|  |  |  | 999 DECLINE TO ANSWER |
| 1. J_MHOPEWELL |  | I trust my future will turn out well. | 1 AGREE |
|  |  |  | 2 DISAGREE |
|  |  |  | 998 DON'T KNOW |
|  |  |  | 999 DECLINE TO ANSWER |
| 1. J_MDEPRES |  | I am frequently stressed or depressed because of not having enough work or income. | 1 AGREE |
|  |  |  | 2 DISAGREE |
|  |  |  | 998 DON'T KNOW |
|  |  |  | 999 DECLINE TO ANSWER |
| 1. J_MASHAME |  | I sometimes feel ashamed that my wife has to work. | 1 AGREE |
|  |  |  | 2 DISAGREE |
|  |  |  | 998 DON'T KNOW |
|  |  |  | 999 DECLINE TO ANSWER |
| 1. J_MRELQUALA |  | PLEASE READ: When two people are married, they usually share both good and bad moments. I would like to ask you some questions about your relationship with your wife. All of your responses will be kept completely confidential and will not be told to anyone. Please tell me if you agree or disagree with the following statement: My spouse and I talk often about problems we are facing in life. | 1 AGREE |
|  |  |  | 2 DISAGREE |
|  |  |  | 998 DON'T KNOW |
|  |  |  | 999 DECLINE TO ANSWER |
| 1. J_MRELQUALC |  | When I have problems my spouse listens to me. | 1 AGREE |
|  |  |  | 2 DISAGREE |
|  |  |  | 998 DON'T KNOW |
|  |  |  | 999 DECLINE TO ANSWER |
| 1. J_MRELQUALD |  | My spouse blames me for things that go wrong. | 1 AGREE |
|  |  |  | 2 DISAGREE |
|  |  |  | 998 DON'T KNOW |
|  |  |  | 999 DECLINE TO ANSWER |
| 1. J_MRELQUALE |  | I feel appreciated by my spouse. | 1 AGREE |
|  |  |  | 2 DISAGREE |
|  |  |  | 998 DON'T KNOW |
|  |  |  | 999 DECLINE TO ANSWER |
| 1. J_MRELQUALF |  | I feel respected even if we disagree. | 1 AGREE |
|  |  |  | 2 DISAGREE |
|  |  |  | 998 DON'T KNOW |
|  |  |  | 999 DECLINE TO ANSWER |
| 1. J_MRELQUALG |  | We are good at solving our differences. | 1 AGREE |
|  |  |  | 2 DISAGREE |
|  |  |  | 998 DON'T KNOW |
|  |  |  | 999 DECLINE TO ANSWER |
| 1. J_MRELQUALH |  | My spouse criticizes my opinions, feelings, or desires. | 1 AGREE |
|  |  |  | 2 DISAGREE |
|  |  |  | 998 DON'T KNOW |
|  |  |  | 999 DECLINE TO ANSWER |
| 1. J_MRELQUALI |  | My spouse shows love and affection for me. | 1 AGREE |
|  |  |  | 2 DISAGREE |
|  |  |  | 998 DON'T KNOW |
|  |  |  | 999 DECLINE TO ANSWER |
| 1. J_MRELQUALJ |  | My spouse and I quarrel frequently | 1 AGREE |
|  |  |  | 2 DISAGREE |
|  |  |  | 998 DON'T KNOW |
|  |  |  | 999 DECLINE TO ANSWER |
| **K. CHILDHOOD EXPERIENCES** | | | |
| 1. K_MCHILDHSHA |  | Now I am going to ask you some questions about your childhood and your parents. When you were a child, how often did your father do the following tasks: Prepare food | 1 NEVER |
|  |  |  | 2 SOMETIMES |
|  |  |  | 3 OFTEN |
|  |  |  | 998 DON'T KNOW |
|  |  |  | 999 DECLINE TO ANSWER |
| 1. K_MCHILDHSHB |  | Clean the house | 1 NEVER |
|  |  |  | 2 SOMETIMES |
|  |  |  | 3 OFTEN |
|  |  |  | 998 DON'T KNOW |
|  |  |  | 999 DECLINE TO ANSWER |
| 1. K_MCHILDHSHC |  | Wash clothes | 1 NEVER |
|  |  |  | 2 SOMETIMES |
|  |  |  | 3 OFTEN |
|  |  |  | 998 DON'T KNOW |
|  |  |  | 999 DECLINE TO ANSWER |
| 1. K_MCHILDHSHD |  | Take care of you or your siblings | 1 NEVER |
|  |  |  | 2 SOMETIMES |
|  |  |  | 3 OFTEN |
|  |  |  | 998 DON'T KNOW |
|  |  |  | 999 DECLINE TO ANSWER |
| 1. K_MCHILDHSHE |  | Shop for household items | 1 NEVER |
|  |  |  | 2 SOMETIMES |
|  |  |  | 3 OFTEN |
|  |  |  | 998 DON'T KNOW |
|  |  |  | 999 DECLINE TO ANSWER |
| 1. K_MCHILDHSHF |  | Fetch water | 1 NEVER |
|  |  |  | 2 SOMETIMES |
|  |  |  | 3 OFTEN |
|  |  |  | 998 DON'T KNOW |
|  |  |  | 999 DECLINE TO ANSWER |
| 1. K_MCHILDDECIS |  | Thinking about your childhood, who had the final word in your household about making major household purchases (for example, buying livestock)? | 1 Father |
|  |  |  | 2 Mother |
|  |  |  | 3 Father and mother JOINTLY |
|  |  |  | 4 Grandmother |
|  |  |  | 5 Grandfather |
|  |  |  | 6 Other wife of father |
|  |  |  | 997 OTHER |
|  |  |  | 998 DON'T KNOW |
|  |  |  | 999 DECLINE TO ANSWER |
| 1. K_MCHWITNES |  | Now some statements will be read to you, and we would like to know whether and how often each of the following things happened to you before you got married. Did they NEVER happen, did they happen only ONCE, did they happen TWO TO TEN TIMES, or did they happen OFTEN? Before I was married, I saw or heard my mother being beaten by my father or another male relative. | 1 NEVER |
|  |  |  | 2 ONLY ONCE |
|  |  |  | 3 TWO TO TEN TIMES |
|  |  |  | 4 OFTEN |
|  |  |  | 998 DON'T KNOW |
|  |  |  | 999 DECLINE TO ANSWER |
| 1. K_MCHABUSEA |  | Before I was married, I was spanked or slapped by my parents in the home. | 1 NEVER |
|  |  |  | 2 ONLY ONCE |
|  |  |  | 3 TWO TO TEN TIMES |
|  |  |  | 4 OFTEN |
|  |  |  | 998 DON'T KNOW |
|  |  |  | 999 DECLINE TO ANSWER |
| 1. K_MCHABUSEB |  | Before I was married,  I was beaten at home with a belt, stick, whip or another hard object. | 1 NEVER |
|  |  |  | 2 ONLY ONCE |
|  |  |  | 3 TWO TO TEN TIMES |
|  |  |  | 4 OFTEN |
|  |  |  | 998 DON'T KNOW |
|  |  |  | 999 DECLINE TO ANSWER |
| 1. K_MCHABUSEC |  | Before I was married, someone touched my intimate parts when I did not want them to. | 1 NEVER |
|  |  |  | 2 ONLY ONCE |
|  |  |  | 3 TWO TO TEN TIMES |
|  |  |  | 4 OFTEN |
|  |  |  | 998 DON'T KNOW |
|  |  |  | 999 DECLINE TO ANSWER |
| 1. K_MCHABUSED |  | Before I was married, I had sex with someone because I was threatened, frightened, or forced. | 1 NEVER |
|  |  |  | 2 ONLY ONCE |
|  |  |  | 3 TWO TO TEN TIMES |
|  |  |  | 4 OFTEN |
|  |  |  | 998 DON'T KNOW |
|  |  |  | 999 DECLINE TO ANSWER |
| 1. K_MCHABUSEE | If _MSCH=1 (household survey) | Before I was married, I was beaten or physically punished at school by a teacher . | 1 NEVER |
|  |  |  | 2 ONLY ONCE |
|  |  |  | 3 TWO TO TEN TIMES |
|  |  |  | 4 OFTEN |
|  |  |  | 998 DON'T KNOW |
|  |  |  | 999 DECLINE TO ANSWER |
| **L. GENDER BELIEFS** | | | |
| 1. L_MBOYSCH |  | [INTRO ON SAME PAGE AS QUESTION] Now I will read some statements about relations between men and women in society. Please feel free to answer in a way that reflects what you truly believe -- there are no right or wrong answers. For the following statements, please state whether you AGREE or DISAGREE. [SEPARATION] If resources are scarce, it is more important to educate sons than daughters. | 1 AGREE |
|  |  |  | 2 DISAGREE |
|  |  |  | 998 DON'T KNOW |
|  |  |  | 999 DECLINE TO ANSWER |
| 1. L_MBOYFREE |  | Boys should have more free time than girls. | 1 AGREE |
|  |  |  | 2 DISAGREE |
|  |  |  | 998 DON'T KNOW |
|  |  |  | 999 DECLINE TO ANSWER |
| 1. L_MBOYRESP |  | Boys are responsible for the behavior of their sisters. | 1 AGREE |
|  |  |  | 2 DISAGREE |
|  |  |  | 998 DON'T KNOW |
|  |  |  | 999 DECLINE TO ANSWER |
| 1. L_MBOYFOOD |  | If there is limited food, the food should be given to the men and boy children first. | 1 AGREE |
|  |  |  | 2 DISAGREE |
|  |  |  | 998 DON'T KNOW |
|  |  |  | 999 DECLINE TO ANSWER |
| 1. L_MGEMMHELPCH |  | Men should not bathe, feed or otherwise take care of children. | 1 AGREE |
|  |  |  | 2 DISAGREE |
|  |  |  | 998 DON'T KNOW |
|  |  |  | 999 DECLINE TO ANSWER |
| 1. L_MGEMOBEY |  | A woman should obey her husband in all things. | 1 AGREE |
|  |  |  | 2 DISAGREE |
|  |  |  | 998 DON'T KNOW |
|  |  |  | 999 DECLINE TO ANSWER |
| 1. L_MGEMVIOTOL |  | A woman should tolerate violence to keep her family together. | 1 AGREE |
|  |  |  | 2 DISAGREE |
|  |  |  | 998 DON'T KNOW |
|  |  |  | 999 DECLINE TO ANSWER |
| 1. L_MGEMROLA |  | A woman’s most important role is to take care of the home and cook for the family. | 1 AGREE |
|  |  |  | 2 DISAGREE |
|  |  |  | 998 DON'T KNOW |
|  |  |  | 999 DECLINE TO ANSWER |
| 1. L_MGEMROLB |  | A man should have the final word about decisions in the home. | 1 AGREE |
|  |  |  | 2 DISAGREE |
|  |  |  | 998 DON'T KNOW |
|  |  |  | 999 DECLINE TO ANSWER |
| 1. L_MGEMROLC |  | More rights for women mean that men lose out. | 1 AGREE |
|  |  |  | 2 DISAGREE |
|  |  |  | 998 DON'T KNOW |
|  |  |  | 999 DECLINE TO ANSWER |
| 1. L_MGEMROLD |  | We have already achieved equality between women and men in society. | 1 AGREE |
|  |  |  | 2 DISAGREE |
|  |  |  | 998 DON'T KNOW |
|  |  |  | 999 DECLINE TO ANSWER |
| 1. L_MGEMROLE |  | There are times when a woman deserves to be beaten. | 1 AGREE |
|  |  |  | 2 DISAGREE |
|  |  |  | 998 DON'T KNOW |
|  |  |  | 999 DECLINE TO ANSWER |
| 1. L_MGEMROLF |  | I think it is shameful when men engage in caring for children or other domestic work. | 1 AGREE |
|  |  |  | 2 DISAGREE |
|  |  |  | 998 DON'T KNOW |
|  |  |  | 999 DECLINE TO ANSWER |
| 1. L_MGEMROLG |  | If another man in my community insults me, I will defend my reputation, with force if I have to. | 1 AGREE |
|  |  |  | 2 DISAGREE |
|  |  |  | 998 DON'T KNOW |
|  |  |  | 999 DECLINE TO ANSWER |
| 1. L_MGEMROLH |  | Giving baths to children, changing children’s clothes, and feeding children are the mother’s responsibility. | 1 AGREE |
|  |  |  | 2 DISAGREE |
|  |  |  | 998 DON'T KNOW |
|  |  |  | 999 DECLINE TO ANSWER |
| 1. L_MGEMROLI |  | A woman should never question her husband’s decisions even if she disagrees with them. | 1 AGREE |
|  |  |  | 2 DISAGREE |
|  |  |  | 998 DON'T KNOW |
|  |  |  | 999 DECLINE TO ANSWER |
| 1. L_MGEMROLJ |  | Women are too emotional to be leaders. | 1 AGREE |
|  |  |  | 2 DISAGREE |
|  |  |  | 998 DON'T KNOW |
|  |  |  | 999 DECLINE TO ANSWER |
| 1. L_MGEMROLK |  | It is natural and right that men have more power than women in the family. | 1 AGREE |
|  |  |  | 2 DISAGREE |
|  |  |  | 998 DON'T KNOW |
|  |  |  | 999 DECLINE TO ANSWER |
| 1. L_MGEMROLL |  | If a man cooks or cleans it is shameful for his wife. | 1 AGREE |
|  |  |  | 2 DISAGREE |
|  |  |  | 998 DON'T KNOW |
|  |  |  | 999 DECLINE TO ANSWER |
| 1. L_MGEMROLM |  | A woman should have the right to end a marriage through divorce. | 1 AGREE |
|  |  |  | 2 DISAGREE |
|  |  |  | 998 DON'T KNOW |
|  |  |  | 999 DECLINE TO ANSWER |
| 1. L_MGEMROLN |  | My only role for caring for my children is as their financial provider. | 1 AGREE |
|  |  |  | 2 DISAGREE |
|  |  |  | 998 DON'T KNOW |
|  |  |  | 999 DECLINE TO ANSWER |
| 1. L_MGEMROLO |  | I wish I spent more time with my children | 1 AGREE |
|  |  |  | 2 DISAGREE |
|  |  |  | 998 DON'T KNOW |
|  |  |  | 999 DECLINE TO ANSWER |
| 1. L_MGEMWCHILD |  | Only when a woman has a child is she a real woman. | 1 AGREE |
|  |  |  | 2 DISAGREE |
|  |  |  | 998 DON'T KNOW |
|  |  |  | 999 DECLINE TO ANSWER |
| 1. L_MGEMMCHILD |  | A real man produces a male child. | 1 AGREE |
|  |  |  | 2 DISAGREE |
|  |  |  | 998 DON'T KNOW |
|  |  |  | 999 DECLINE TO ANSWER |
| 1. L_MGEMDECSX |  | It is the man who decides if and when to have sex. | 1 AGREE |
|  |  |  | 2 DISAGREE |
|  |  |  | 998 DON'T KNOW |
|  |  |  | 999 DECLINE TO ANSWER |
| 1. L_MGEMREDSX |  | Men are always ready to have sex. | 1 AGREE |
|  |  |  | 2 DISAGREE |
|  |  |  | 998 DON'T KNOW |
|  |  |  | 999 DECLINE TO ANSWER |
| 1. L_MGEMMORSX |  | Men need sex more than women do. | 1 AGREE |
|  |  |  | 2 DISAGREE |
|  |  |  | 998 DON'T KNOW |
|  |  |  | 999 DECLINE TO ANSWER |
| 1. L_MGEMINITSX |  | A woman should not initiate sex. | 1 AGREE |
|  |  |  | 2 DISAGREE |
|  |  |  | 998 DON'T KNOW |
|  |  |  | 999 DECLINE TO ANSWER |
| 1. L_MGEMMARSX |  | A woman who has sex before she marries does not deserve respect. | 1 AGREE |
|  |  |  | 2 DISAGREE |
|  |  |  | 998 DON'T KNOW |
|  |  |  | 999 DECLINE TO ANSWER |
| 1. L_MREFSXREP |  | If a woman refuses to have sex with her husband when he wants her to, he has the right to get angry and reprimand her. | 1 AGREE |
|  |  |  | 2 DISAGREE |
|  |  |  | 998 DON'T KNOW |
|  |  |  | 999 DECLINE TO ANSWER |
| 1. L_MREFSXFIN |  | If a woman refuses to have sex with her husband when he wants her to, he has the right to refuse to give her money or other means of financial support. | 1 AGREE |
|  |  |  | 2 DISAGREE |
|  |  |  | 998 DON'T KNOW |
|  |  |  | 999 DECLINE TO ANSWER |
| 1. L_MREFSXFORC |  | If a woman refuses to have sex with her husband when he wants her to, he has the right to use force and have sex with her even if she doesn’t want to. | 1 AGREE |
|  |  |  | 2 DISAGREE |
|  |  |  | 998 DON'T KNOW |
|  |  |  | 999 DECLINE TO ANSWER |
| 1. L_MJUSTVOUT |  | In your opinion, is a husband justified in hitting or beating his wife in the following situations: If she goes out without telling him? | 1 YES |
|  |  |  | 2 NO |
|  |  |  | 998 DON'T KNOW |
|  |  |  | 999 DECLINE TO ANSWER |
| 1. L_MJUSTVFP |  | In your opinion, is a husband justified in hitting or beating his wife in the following situations: If she uses a family planning method without his permission? | 1 YES |
|  |  |  | 2 NO |
|  |  |  | 998 DON'T KNOW |
|  |  |  | 999 DECLINE TO ANSWER |
| 1. L_MJUSTVARG |  | In your opinion, is a husband justified in hitting or beating his wife in the following situations: If she argues with him? | 1 YES |
|  |  |  | 2 NO |
|  |  |  | 998 DON'T KNOW |
|  |  |  | 999 DECLINE TO ANSWER |
| 1. L_MJUSTVSX |  | In your opinion, is a husband justified in hitting or beating his wife in the following situations: If she refuses to have sex with him? | 1 YES |
|  |  |  | 2 NO |
|  |  |  | 998 DON'T KNOW |
|  |  |  | 999 DECLINE TO ANSWER |
| 1. L_MJUSTVBRN |  | In your opinion, is a husband justified in hitting or beating his wife in the following situations: If she burns his food? | 1 YES |
|  |  |  | 2 NO |
|  |  |  | 998 DON'T KNOW |
|  |  |  | 999 DECLINE TO ANSWER |
| 1. L_MBYSTAND |  | What did you do the last time when you saw, heard, witnessed, or otherwise knew that your male friend was using physical violence against his spouse? | 1 I DID NOTHING |
|  |  |  | 2 DIRECTLY INTERVENED OR ASKED THE MAN’S FAMILY TO INTERVENE |
|  |  |  | 3 CONTACTED INFORMAL/RELIGIOUS LEADER |
|  |  |  | 4 CONTACTED POLICE |
|  |  |  | 5 CONTACTED COMMUNITY LEADER |
|  |  |  | 6 Have never had this experience |
|  |  |  | 997 OTHER |
|  |  |  | 998 DON'T KNOW |
|  |  |  | 999 DECLINE TO ANSWER |
| 1. L_MVIOPUNISH |  | Are there any consequences for men who are violent against their wife? | 1 YES |
|  |  |  | 2 NO |
|  |  |  | 998 DON'T KNOW |
|  |  |  | 999 DECLINE TO ANSWER |
| **M. SOCIAL NORMS** | | | |
| 1. M_MSNGEMA |  | People in my village think that a woman’s most important role is to take care of the home and cook for the family . | 1 AGREE |
|  |  |  | 2 DISAGREE |
|  |  |  | 998 DON'T KNOW |
|  |  |  | 999 DECLINE TO ANSWER |
| 1. M_MSNGEMB |  | People in my village think that a man should have the final word about decisions in the home. | 1 AGREE |
|  |  |  | 2 DISAGREE |
|  |  |  | 998 DON'T KNOW |
|  |  |  | 999 DECLINE TO ANSWER |
| 1. M_MSNGEMC |  | People in my village think that there are times when a woman deserves to be beaten. | 1 AGREE |
|  |  |  | 2 DISAGREE |
|  |  |  | 998 DON'T KNOW |
|  |  |  | 999 DECLINE TO ANSWER |
| 1. M_MSNGEMD |  | People in my village think that it is shameful when men engage in caring for children or other domestic work. | 1 AGREE |
|  |  |  | 2 DISAGREE |
|  |  |  | 998 DON'T KNOW |
|  |  |  | 999 DECLINE TO ANSWER |
| 1. M_MSNGEME |  | People in my village think that giving baths to children, changing children’s clothes, and feeding children are the mother’s responsibility | 1 AGREE |
|  |  |  | 2 DISAGREE |
|  |  |  | 998 DON'T KNOW |
|  |  |  | 999 DECLINE TO ANSWER |
| 1. M_MSNGEMF |  | People in my village think that a woman should never question her husband’s decisions even if she disagrees with them | 1 AGREE |
|  |  |  | 2 DISAGREE |
|  |  |  | 998 DON'T KNOW |
|  |  |  | 999 DECLINE TO ANSWER |
| 1. M_MSNGEMG |  | People in my village think that It is natural and right that men have more power than women in the family | 1 AGREE |
|  |  |  | 2 DISAGREE |
|  |  |  | 998 DON'T KNOW |
|  |  |  | 999 DECLINE TO ANSWER |
| 1. M_MSNGEMH |  | People in my village think that if a man cooks or cleans it is shameful for his wife | 1 AGREE |
|  |  |  | 2 DISAGREE |
|  |  |  | 998 DON'T KNOW |
|  |  |  | 999 DECLINE TO ANSWER |
| 1. M_MSNGEMI |  | People in my village expect that girls decide when and who to marry. | 1 AGREE |
|  |  |  | 2 DISAGREE |
|  |  |  | 998 DON'T KNOW |
|  |  |  | 999 DECLINE TO ANSWER |
| 1. M_MCOMTALKFP |  | People in this community expect me to talk with my wife about doing something to space or delay pregnancy. | 1 AGREE |
|  |  |  | 2 DISAGREE |
|  |  |  | 998 DON'T KNOW |
|  |  |  | 999 DECLINE TO ANSWER |
| 1. M_MCOMDECCH |  | People in my village expect me to decide how many children my family will have. | 1 AGREE |
|  |  |  | 2 DISAGREE |
|  |  |  | 998 DON'T KNOW |
|  |  |  | 999 DECLINE TO ANSWER |
| 1. M_MCOMPWR |  | People in my village will think I'm more powerful if I have more children. | 1 AGREE |
|  |  |  | 2 DISAGREE |
|  |  |  | 998 DON'T KNOW |
|  |  |  | 999 DECLINE TO ANSWER |
| 1. M_MCOMWEAK |  | People in my community will think I am weak if I help my wife with household chores or taking care of my kids. | 1 AGREE |
|  |  |  | 2 DISAGREE |
|  |  |  | 998 DON'T KNOW |
|  |  |  | 999 DECLINE TO ANSWER |
| 1. M_MCOMBEAT |  | CHANGED ORDER People in this community expect that a husband will beat his wife | 1 AGREE |
|  |  |  | 2 DISAGREE |
|  |  |  | 998 DON'T KNOW |
|  |  |  | 999 DECLINE TO ANSWER |
| 1. M_MCOMMENFP |  | CHANGED ORDER What would be the reaction of most men in your community if their wife used a contraceptive method or tried in any way to delay or avoid getting pregnant? They would think it is: | 1 good and help her |
|  |  |  | 2 good but do nothing |
|  |  |  | 3 bad, but do nothing |
|  |  |  | 4 bad and try to stop her |
|  |  |  | 997 OTHER |
|  |  |  | 998 DON'T KNOW |
|  |  |  | 999 DECLINE TO ANSWER |
| 1. M_MMENPERSFP |  | What would be the reaction of most men in your community if their wife wanted to use a family planning method to delay or space births? They would think it is: | 1 good and help her |
|  |  |  | 2 good but do nothing |
|  |  |  | 3 bad, but do nothing |
|  |  |  | 4 bad and try to stop her |
|  |  |  | 997 OTHER |
|  |  |  | 998 DON'T KNOW |
|  |  |  | 999 DECLINE TO ANSWER |
